# A Bayesian Network Approach to Explainable Reinforcement Learning with Distal Information

**DOI:** 10.3390/s23042013

**Published:** 2023-02-10

**Authors:** Rudy Milani, Maximilian Moll, Renato De Leone, Stefan Pickl

**Affiliations:** 1 Faculty of Computer Science, Universitaet der Bundeswehr Muenchen, Werner-Heisenberg-Weg 39, 85577 Neubiberg, Germany; 2 School of Science and Technology, University of Camerino, via Madonna delle Carceri 9, 62032 Camerino, Italy

**Keywords:** Explainable Reinforcement Learning, Bayesian Network, model-free methods, causal explanation, human study

## Abstract

Nowadays, Artificial Intelligence systems have expanded their competence field from research to industry and daily life, so understanding how they make decisions is becoming fundamental to reducing the lack of trust between users and machines and increasing the transparency of the model. This paper aims to automate the generation of explanations for model-free Reinforcement Learning algorithms by answering “why” and “why not” questions. To this end, we use Bayesian Networks in combination with the NOTEARS algorithm for automatic structure learning. This approach complements an existing framework very well and demonstrates thus a step towards generating explanations with as little user input as possible. This approach is computationally evaluated in three benchmarks using different Reinforcement Learning methods to highlight that it is independent of the type of model used and the explanations are then rated through a human study. The results obtained are compared to other baseline explanation models to underline the satisfying performance of the framework presented in terms of increasing the understanding, transparency and trust in the action chosen by the agent.

## 1. Introduction

In recent years, the increase in the application of Machine Learning (ML) techniques, due to the availability of faster computers and the need to analyze a large amount of data, has renewed interest in such algorithms. However, ML algorithms sometimes lack explainability and transparency, which is fundamental for being trusted by users [1,2,3,4]. In particular, this is still an unsolved problem for Reinforcement Learning (RL), especially for model-free methods. This kind of techniques estimates the optimal policy without using or evaluating the dynamics of the environment. For this reason, they are difficult to explain, but, on the other hand, these RL techniques have applications in sensitive fields such as medicine [5,6] and finance [7], where choosing one action over another can lead to substantial differences in outcome [4]. Overall, the problem of generating explanations for model-free RL is very important and interesting.

This paper aims to introduce improvements to an existing method [8,9] for generating an explanation for model-free RL agents. The general idea is to predict the information at the next time steps and the most important aspects of a particular problem using a combination of Bayesian Networks (BNs) and Recurrent Neural Networks (RNNs). In detail, we focused on creating explanations for both the actual and counterfactual questions, i.e., respectively, “why” and “why not”. Consequently, this model is computationally evaluated in three RL benchmark environments (two continuous and one discrete) using different trained RL agents and the results are compared to the literature. Lastly, the explanations generated are comparatively rated in a human study together with baseline approaches to check their quality.

The paper is structured as follows: first, the theoretical background is introduced; then, the related works, and in particular Madumal et al.’s method [8,9], are presented. In the following section, our methodology is explained in detail and the experimental results are shown. In the end, a comparison of the results is provided and a discussion about the highlights of this research and future works is presented.

## 2. Background

In this section, the basic theoretical knowledge is discussed. First, RL will be briefly introduced. Then, BNs are described with the NOTEARS algorithm that allows us to obtain the directed acyclic graph.

### 2.1. Reinforcement Learning

RL is one of the three learning paradigms in ML next to Supervised and Unsupervised Learning. In RL, there is an agent, which is the learner and decision-maker: it has to understand how to use the information of the environment to choose the best action. Everything that interacts with the agent is called the environment. The agent can influence and change the environment through its actions while the environment gives rewards whose sum must be maximized over time.

**Figure 1:**
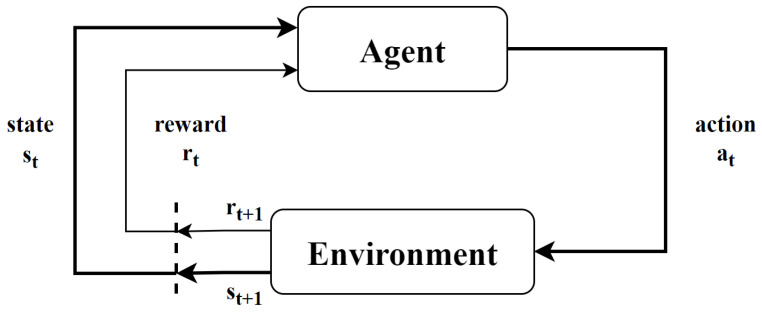
Agent–environment interaction in RL [10].

In more detail, at each discrete time step t=0,1,2… the agent receives the actual state $s_{t}\in S\subseteq R^{n}$ from the environment and decides on an action $a_{t}\in A\subseteq R^{m}$, where S and A are the state and action spaces, respectively. As a consequence of the action, the agent will receive a numerical reward $r_{t}\in R$ and the environment will update to a new state $s_{t+1}$. This procedure is shown in Figure 1. The agent has to choose actions to maximize the sum of rewards $R_{t}=\sum_{i=0}^{\infty }\gamma^{i}r_{t+i+1}$, where γ∈[0,1) is a discounting factor. An additional characterizing property is that the probability of each value for $s_{t}$ and $r_{t}$ depends only on the state $s_{t-1}$ and action $a_{t-1}$ and not on earlier states and actions. This property is called the Markov property and characterizes the Markov Decision Process (MDP) framework. An MDP is a tuple (S,A,T,R), where S and A are previously defined, $T(s_{t},a_{t},s_{t+1})$ is the probability of transitioning from state $s_{t}$ to state $s_{t+1}$ after taking action $a_{t}$ and R:S×A→R is the reward function such that $R\left(s_{t},a_{t}\right)=r_{t+1}$. Another option is to consider the reward function using the transition probability: $R\left(s,a,s^{\prime }\right)=E\left[r_{t+1}|s_{t}=s,a_{t}=a,s_{t+1}=s^{\prime }\right]$. The MDP is the basis of the RL algorithms we will further explore in the following: the *model-free* methods. These algorithms do not need any knowledge of the transitional probability distribution T of the environment, but they consider only the Q-function $Q_{\pi }\left(s,a\right)=E_{\pi }\left[\sum_{k=0}^{\infty }\gamma^{k}r_{t+k+1}|s_{t}=s,a_{t}=a\right]$ under the policy π, which is defined simply as a distribution over actions given states: $\pi \left(a|s\right)=T\left(a_{t}=a|s_{t}=s\right)$. In particular, the methods used in this work are Sarsa, Deep Q-Network (DQN) and Policy Gradient.

### 2.2. Sarsa

The first RL algorithm we will study is *Sarsa*, a model-free and on-policy method: a policy has to be followed to apply this algorithm. The first step of the Sarsa method [10] is to learn the action value function $Q_{\pi }\left(s,a\right)$. At the beginning of the learning process, a Q-table ($Q\left(s_{t},a_{t}\right)\approx Q_{\pi }\left(s_{t},a_{t}\right)$) is initialized as a matrix of zeros. The dimensions of this table are defined by the number of states and actions |S|×|A|. The agent looks at this matrix in order to determine its next action, i.e., using an ϵ-greedy strategy, where ϵ is a fixed constant that represents the probability of choosing a random action. After taking an action, the Q-table is updated considering the following rule:

(1) $$Q\left(s_{t},a_{t}\right)\leftarrow Q\left(s_{t},a_{t}\right)+\alpha [r_{t+1}+\gamma Q\left(s_{t+1},a_{t+1}\right)-Q\left(s_{t},a_{t}\right)],$$

where α∈[0,1] is the learning rate and γ∈[0,1] is the discounting factor. This update is carried out after each step until a terminal state is reached. As we can see from the updating Formula (1), at each iteration a quintuple of events $\left(s_{t},a_{t},r_{t+1},s_{t+1},a_{t+1}\right)$ is needed. This quintuple gives the name *Sarsa* to this algorithm.

### 2.3. DQN

Before introducing DQN, we need to give some basic notions on *Q-learning*. The *Q-learning* algorithm [11] utilizes the same basic ideas of the *Sarsa* algorithm. We start with a Q-table initialized at zero and, at each step, the Q-value [10] is updated using now the following rule:

(2) $$Q\left(s_{t},a_{t}\right)\leftarrow Q\left(s_{t},a_{t}\right)+\alpha \left[r_{t+1}+\gamma \max_{a\in A}Q\left(s_{t+1},a\right)-Q\left(s_{t},a_{t}\right)\right],$$

where as always α is the learning rate and γ the discounting factor. The DQN agent is a combination of the previous RL method and a class of Artificial Neural Networks (ANNs) called Deep Neural Networks (DNNs). The use of ANNs is justified by the fact that when a ReLU function is utilized as an activation function, the ANN is an universal function approximator [12]. Therefore such ANNs can well approximate any Lebesgue integrable function with respect to $L^{1}$ distance if the network’s depth is sufficient enough. In this particular case, the approximate value function is $Q(s,a;\theta_{i})$ where $\theta_{i}$ are the parameters (weight of the arcs) of the Q-network at iteration *i*. We refer to the neural network function approximator as the Q-network. With this structure, the agent can achieve a high level of performance on different problems without using different specific feature sets. At each time step *t*, the agent’s experience $e_{t}=\left(s_{t},a_{t},r_{t},s_{t+1}\right)$ is stored in a dataset $D_{t}=e_{1},\cdots ,e_{t}$. The Q-learning updates are applied during the learning on minibatches of experience $\left(s,a,r,s^{\prime }\right)∼U\left(D\right)$, taken uniformly at random from the memory *D*. Then, the loss function used for the updating is defined as follows:

(3) $$L_{i}\left(\theta_{i}\right)=E_{s,a,r,s^{\prime }}\left[{\left(y_{i}-Q\left(s,a;\theta_{i}\right)\right)}^{2}\right],$$

where $y_{i}=r+\gamma \max_{a^{\prime }\in A}Q\left(s^{\prime },a^{\prime };\theta_{i-1}\right)$ is the target at iteration *i*. By differentiating the loss function with respect to the parameters, we obtain:

(4) $$\nabla_{\theta_{i}}L_{i}\left(\theta_{i}\right)=E_{s,a,r,s^{\prime }}\left[(r+\gamma \max_{a^{\prime }\in A}Q\left(s^{\prime },a^{\prime };\theta_{i-1}\right)-Q\left(s,a;\theta_{i}\right))\nabla_{\theta_{i}}Q\left(s,a;\theta_{i}\right)\right].$$

Instead of computing the full expectations of the gradient, it is often better to optimize the loss function by stochastic gradient descent. This approach has several advantages over standard Q-learning. The most important is that each step of the experience is used to update many different weights and that also increases the efficiency of the method. Another interesting aspect is that randomizing the samples allows for breaking the correlations between consecutive samples. However, there are also flaws. It is easy to get stuck in loops near a poor local minimum or even diverge. However, using experience replay, it is possible to avoid oscillations or divergence in the parameters.

### 2.4. Policy Gradient

All the methods previously presented are action-value methods: they learn the values of the action and they choose the one that maximizes the estimated action value. In this section, different kinds of processes are analyzed that learn a parametrized policy without directly using any value function. Let $\theta \in R^{d^{\prime }}$ be the set of parameters that parametrize the policy $\pi_{\theta }$ (we will use π for brevity). Thus

(5) $$\pi \left(a|s,\theta \right)=P\left\{a_{t}=a|s_{t}=s,\theta_{t}=\theta \right\}$$

is the probability of taking action *a* at time *t* given the state *s* at time *t* and parameters θ. Then, a scalar performance measure J(θ) has to be maximized with respect to the policy parameter. Using the gradient ascent method, we obtain:

(6) $$\theta_{t+1}=\theta_{t}+\alpha \nabla J\left(\theta_{t}\right),$$

where $\nabla J\left(\theta_{t}\right)\in R^{d^{\prime }}$ is the gradient of the performance measure with respect to the policy parameter θ. This is the general idea of the methods known as Policy Gradient methods [13]. As a performance function, the expected return following a parametrized policy can be utilized:

(7) $$J\left(\theta \right)=E_{\pi }\left[R(\tau )\right],$$

where R(τ) is the total return following the trajectory τ. The challenge here is to calculate the gradient of an expectation. This calculation is the result of the Policy Gradient Theorem [13], which states:

(8) $$\nabla J\left(\theta \right)=E_{\pi }[R\left(\tau \right)(\sum_{t=1}^{T}\nabla \log\left(\pi \left(a_{t}|s_{t},\theta \right)\right))].$$

An important aspect that has not been described yet is how we can parametrize our policy π. The only condition that we have to assure is the differentiability with respect to the parameter θ. Considering every policy π(a|s,θ) for which ∇π(a|s,θ) is finite ∀s∈S,∀a∈A and $\theta \in R^{d^{\prime }}$ is a good choice.

### 2.5. Bayesian Network

A BN B=(G,P) consists of a *Directed Acyclic Graph* (DAG) G=(V,E) containing vertices *V* and edges *E* and a set of conditional probability distributions *P*. A *DAG* is a graph where all the edges point in a specific direction and there are no cycles (it is not possible to start from any node and return to it following the direction of the edges). Each vertex in G=(V,E) corresponds to a feature and the edges represent a dependency between two features. This graph *G* is also called the *structure* of the BN. Another interesting domain is the one relative to Dynamic Bayesian Networks (DBNs). A DBN deals with variables in a BN that change with time. For the definition of a DBN, we need two structures: a prior network that represents the interactions between variables at time t=0 and a second transition network that can illustrate how each variable is conditioned by the one from the previous (or same) time slices [14].

### 2.6. NOTEARS Algorithm

In this subsection, the principal features of the NOTEARS algorithm are introduced; for major details, we refer to [15]. The task of learning DAGs from data is an extremely complex problem since it is an NP-hard problem [16] and NP-complete [17]. For this reason, it is helpful to describe this combinatorial problem using a particular smooth function as follows:

(9) minW∈Rd×dF(W)subjecttoh(W)=0,

where

$$F\left(W\right)=l\left(W;X\right)+{\lambda ∥W∥}_{1}=\frac{1}{2n}{∥X-XW∥}_{F}^{2}+\lambda {∥W∥}_{1},$$

and

$$h\left(W\right)=tr\left(e^{W\circ W}\right)-d.$$

Here, *W* is the weighted adjacency matrix, which is a particular case of adjacency matrices where, instead of 0−1, there are the weights $w_{ij}$ of the arcs between nodes. In addition, $F:R^{d\times d}\to R$ is a score function and $h:R^{d\times d}\to R$ is a smooth function whose level set at zero characterizes directed acyclic graphs. The algorithm proposed by [15] for solving (9) consists of three steps:

1. Writing the constrained problem as a sequence of unconstrained subproblems;
2. Solving the subproblems: first the primal one and then dual ascent is applied. This process is iterated until we obtain that the constraint is verified with a tolerance of $\epsilon_{M}$;
3. Utilizing thresholding: eliminating all the edges that present a weight lower than a threshold parameter ϵ.

All these steps compose the NOTEARS algorithm, which is summarized in Algorithm 1. The computational complexity of this method is cubic in the number of nodes, although the constant is small for sparse matrices and it outperforms many of the existing algorithms when the in-degree is large. However, there are no studies on the worst-case iteration complexity since in practice not many (t∼10) iterations are required [15]. In the end, although the method guarantees to find only stationary points, the scores attained by the solutions found are comparable to the global optimal scores [15].
**Algorithm 1** NOTEARS algorithm [15]**Require:** Initial guess $\left(W_{0},\alpha_{0}\right)$, progress rate c∈(0,1), tolerance $\epsilon_{M}>0$, threshold ϵ>0.  1: **for** 
t=0,1,⋯,∞:
**do**  2:       **Solve primal problem**:  3:       $W_{t+1}\leftarrow$ argmin $\left(L^{\rho }\left(W,\alpha_{t}\right)\right)$ with ρ s.t. $h\left(W_{t+1}\right)<c\cdot h\left(W_{t}\right).$  4:       **Dual ascent**:  5:       $\alpha_{t+1}\leftarrow \alpha_{t}+\gamma h\left(W_{t+1}\right)$  6:       **if** $h\left(W_{t+1}\right)<\epsilon_{M}$ **then**  7:           $W_{ECP}^{*}\leftarrow W_{t+1}$  8:           **break**  9:       **end if**  10: **end for**  11: **Thresholding**:  12: $W^{*}\leftarrow W_{ECP}^{*}\circ 1_{\left[|W_{ECP}^{*}|>\epsilon \right]}$  13: **Return** 
$W^{*}$

### 2.7. Explainable Reinforcement Learning Terminology

In recent years, with the explosion of Explainable Reinforcement Learning (XRL) usage in practice, the ambiguity in the vocabulary considered in different research in this field has increased [18,19]. This fact creates huge problems if comparing papers adopting contrasting terminologies. The most relevant example of ambiguity is in the definition of the central concept of an explainable method [20]. A recurrent circumstance is, indeed, when authors do not explicitly give the definition of what they consider explainable or interpretable [21], generating in this way more doubt. Although in different papers [18,22,23] explainability is considered as a synonym of interpretability, we recognize these two terms as distinct: a model is qualified as *interpretable* if we can intrinsically understand it without any further developments; while an approach is *explainable* when we apply an external algorithm that can generate a reasonable explanation. Therefore, interpretability can be regarded as a passive and explainability as active characteristic.

However, this is not the only equivocal case in the terminology. Other controversial examples are: transparency, comprehensibility, simulatability and decomposability [19,21,24,25]. In particular, for the purpose of this paper, we focus our attention on the definition of the following concepts:

- Transparency: the ability to give relevant information about a model, e.g., feature relationships;
- Trustworthy: when a user can reasonably be confident in the choices of the algorithm;
- Understandable: if a user can comprehend why the explanation was generated in that way;
- Satisfying: the feeling of having all the information well organized;
- Safe-reliable: being able to constantly behave in the same way;
- Complete: when all the fundamental components are presented in the explanation.

After this brief introduction to the vocabulary adopted in this paper, we can move to the literature review presented in the next section.

## 3. Related Work

In this section, past work on the explanation and interpretability of RL methods is discussed. In particular, we focus first on a general overview of the most important previous publications and later we give the details of the existing method from which we will develop our methodology.

### 3.1. General Overview

The literature that explores the explanation of policies and actions in an MDP context is extensive. In [26], a first attempt to generate explanations for MDP selects and uses relevant variables of states of factored MDPs, chosen by domain experts. Then, attention was brought to the generation of minimal explanations for MDPs using three domain-independent templates [27]. Moreover, policy explanations were created from summarized policies and provided contrastive explanations for counterfactual events [28]. Similarly to our approach, ref. [29] proposes to use DBNs for representing the transition function in factored MDPs, since learning DBNs in factored MDPs is a well known solved problem [30]. It is fundamental for the increased transparency of the machine to be able to create explanations for the counterfactual case, which simulates what could happen under different conditions. These answers will give a detailed explanation of the agent’s behaviour [22,31,32].

Another category of methods for XRL analyzed images of Atari games and constructed counterfactual explanations through a deep generative model, obtaining interesting results in the identification of flawed agents [33]. Other previous works that used images for understanding deep RL agents focused on saliency maps and t-SNE embeddings [34,35,36].

A different approach to XRL is given by reward decomposition into meaningful reward types, to directly compare the kind of action done for a sufficiently minimal explanation [37,38,39]. Similarly, it is possible to decouple the state feature extraction to reduce the search space, accelerate the training and give a better interpretable representation [40]. However, these methods do not use an underlying causal model, which is fundamental for our purposes. Causal models are usually defined by structural equations that can also model counterfactual explanations [41]. In the next subsection, we focus our attention on the *Action-Influence graph* approach which is the starting point for defining our methodology.

### 3.2. Action-Influence Model Approach

In this subsection, we introduce Madumal et al.’s [8,9] method for the generation of explanations in RL using Action-Influence graphs. In these papers, the authors apply different approaches (linear regression, decision tree, multi-layer perceptron, decision policy tree and decision policy tree with fixed depth) to learn the structural equations of the causal model and compare the results between them. The explanations are generated using an Action-Influence graph given by an expert in the domain. In the following, the formal definition of the Action-Influence model is provided.

**Definition** **1** ([9])**.** *An Action-Influence model is a tuple $\left(S_{a},F\right)$, where $S_{a}$ is a tuple (U,V,R,A) in which U is the set of the exogenous variables, V the set of endogenous variables, R is a function that denotes the range of values of every variable in U∪V and A is the set of actions and F is the set of structural equations.*

Due to this graph, it is possible to understand how the agent’s actions interact with the environment and to explain the relationship between each feature. Before giving the definitions of the minimal explanation, we need to introduce the concept of *instantiation* [9] for both the actual and counterfactual scenarios.

**Definition** **2** ([9])**.** *The actual instantiation of a model M is defined as $M_{V\leftarrow S}$, where S is the vector of state variable values from an MDP and V gives the set of the endogenous variables of the action influence model.*

**Definition** **3** ([9])**.** *The counterfactual instantiation of a model M for a counterfactual action B is defined as $M_{Z\leftarrow S_{Z}}$, where Z gives the instantiation of all the predecessor variables of action B with current state values and the instantiation of all successor nodes (of B) of the causal chain by forwarding simulation.*

Now, it is possible to understand how *minimally complete explanations* are generated. For the answer to the “why” question, the authors from [8] provide the following definition that uses a decision tree as a predictor.

**Definition** **4** ([8])**.** *Given the set of decision nodes $X_{d}=x_{d}$ for the action a from a decision tree T, we define a minimally complete explanation for a “why” question as a pair $\left(X_{r}=x_{r},X_{n}=x_{n}\right)$, in which $X_{r}$ is the vector of the reward variables reached by following the causal chain of the graph to sink nodes; $X_{n}$ is such that $X_{n}$ is the maximal set of variables in which $X_{n}=\left(X_{a}=x_{a}\right)\cap \left(X_{d}=x_{d}\right)$, where $X_{a}$ is the set of intermediate nodes of the causal chain of action a, with $x_{r},x_{a}$ and $x_{d}$ giving the values under the actual instantiation $M_{V\leftarrow S}$.*

For the counterfactual question, they use a different concept based on *minimally complete contrastive explanation*.

**Definition** **5** ([8])**.** *Given the set of decision nodes $X_{d}=x_{d}$ for the action a from a decision tree T, we define a minimally complete contrastive explanation for a “why not” question as a pair $\left(X_{r}=x_{r},X_{con}=x_{con}\right)$, in which $X_{r}$ is the vector of the reward variables reached by following the causal chain of the graph to sink nodes; $X_{con}$ is such that $X_{con}$ is the maximal set of variables in which $X_{con}=\left(X_{b}=x_{b}\right)\cap \left(X_{c}=x_{c}\right)$, where $X_{b}$ is the set of intermediate nodes of the causal chain of the counterfactual action b, $X_{c}$ is the set of the counterfactual decision nodes (generated obtaining the counterfactual action and then traversing the tree) and values $x_{r}$ and $x_{c}$ are contrasted using the actual instantiation $M_{V\leftarrow S}$ and the counterfactual instantiation $M_{Z\leftarrow S_{Z}}$.*

Then, they also added the distal explanation: forecasting the most significant action in that episode through an RNN. For this reason, the explanations must be modified in order to consider the new information obtained.

**Definition** **6** ([8])**.** *Given a minimally complete contrastive explanation, current action a and a prediction model L, a minimally complete distal explanation is a tuple $\left(X_{r}=x_{r},X_{con}=x_{con},a_{d}\right)$, where $X_{r}$ and $X_{con}$ do not change from Definition 5, and $a_{d}$ gives the distal action predicted through L such that $a_{d}\in A\cap A_{c}$, where A is the action set of the agent and $A_{c}$ gives the action set of the causal chain of current action a.*

Hence, for the “why” questions, the explanation is created following the chain of the Action-Influence graph given by the action predicted using one of the previous techniques and the distal action. On the other hand, the counterfactual explanation is produced by comparing the chain given by the counterfactual action with the actual one.

The environment that is used for testing the results on the explanation in [8] is the Starcraft II scenario. Starcraft II is a science fiction real-time strategy video game where two or more players compete against each other. Each person has to develop his base and then defend it or attack and defeat the other players. Due to its high complexity, this game started to be an important benchmark for the creation of competitive AIs that could obtain better results than any other human player [42]. The state variables are *W* worker number, *S* supply depot number, *B* barracks number, *E* enemy location, $A_{n}$ ally unit number, $A_{h}$ ally unit health, $A_{l}$ ally unit location, $D_{u}$ destroyed units and $D_{b}$ destroyed buildings. The set of actions is composed of: $A_{s}$ build supply depot, $A_{b}$ build barracks, $A_{m}$ train offensive unit and $A_{a}$ attack. An example of counterfactual explanation for the question “why not build barracks ($A_{b}$)?” produced by [8] is:

“Because ally unit number ($A_{n}$) is less than the optimal number 18, it is more desirable do the action train marine ($A_{m}$) to enable the action attack ($A_{a}$) as the goal is to have more Destroyed Units ($D_{u}$) and Destroyed buildings ($D_{b}$)”.

In summary, this method begins with a technique for the prediction of the action performed by the agent; then, following the correct chain from the Action-Influence graph and using the distal action obtained from a RNN, the explanation is created. For more information and examples, we refer to [8,9]. This method is the starting point of our approach, but in our case, some changes will be introduced to obtain improvements in the accuracy of the prediction and increase the details of the explanations.

## 4. Our Method

**Figure 2:**
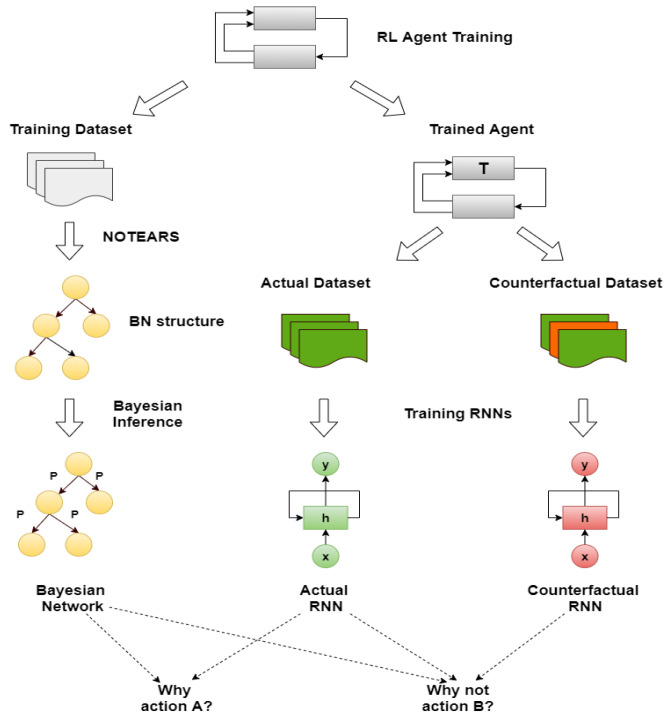
Pipeline of the proposed methodology.

**Figure 3:**
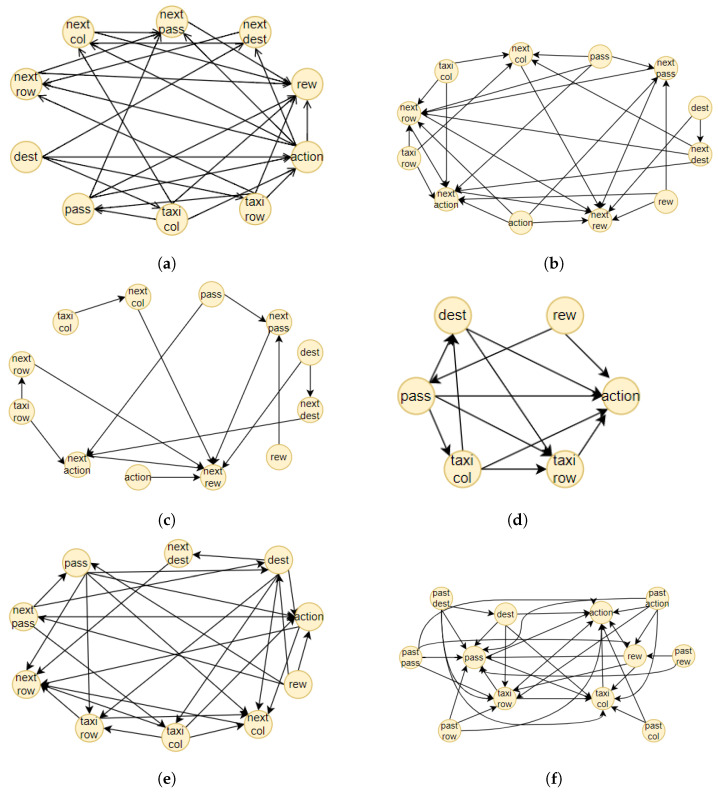
Comparison of causal graphs generated with different algorithms and parameters for Taxi environment. (**a**) BN Graph created by the NOTEARS algorithm with threshold parameter ϵ=0.5. (**b**) DBN Graph created by the DYNOTEARS algorithm with threshold parameter ϵ=0.01. (**c**) DBN created by the DYNOTEARS algorithm with threshold parameter ϵ=0.1. (**d**) Causal graph generated from Direct LiNGAM using only the actual state values. (**e**) Causal graph generated from Direct LiNGAM using both the actual state values and the next ones. (**f**) Causal graph generated from VARLiNGAM with the threshold for the actual relation ϵ=0.01 and for the next time step $\epsilon_{A}=0.1$.

The proposed method consists of four components, illustrated together with the complete pipeline of the methodology in Figure 2. The first one is an RL agent, trained with a model-free method. During the training, the state-action pairs are saved in a dataset, the memory, to build the BN (the second component). The memory is composed of all the actual states, the action chosen by the agent, the next states and the reward obtained. From this dataset, we generated different causal graphs using different algorithms, represented in Figure 3. We decided to adopt our final methodology after a comparison of the different results obtained using approaches well known in the literature. In the first case, Figure 3a, we created a causal structure that is in between a BN and a DBN. In fact, for each of the memory’s characteristics, we consider a node in the BN. To create the structure of this BN, the NOTEARS algorithm, as discussed in Section 2.6, is utilized. The obtained graph is similar to a DBN but it does not present all the features in the next time step. In fact, we do not use any future information for the reward and action. Furthermore, during the last step of the NOTEARS algorithm (the thresholding), we include some assumptions to obtain a more realistic structure. The conditions are the following:

- Causality: all the state features influence their corresponding future values (so no future feature can influence current states or there will be a cycle that is not allowed);
- Stationarity of dependencies between variables: if there is a dependency between features at a one-time step, then it will be in every time step;
- Importance of the action: all the values of the actual state will be used to obtain the action and the action node is connected to all the future features.

These assumptions are justified by the observation of the other graphs. The first two hypotheses come from the DBN since they are fundamental conditions for generating this kind of graph. The third one is, instead, derived from the relations obtained using the Direct LiNGAM [43] and VARLiNGAM [44]. In the last graphs presented in Figure 3e,f, it is possible to see how all the state variables are connected to the action. This is also justified by the fact that the RL agent is trained to consider the state values and choose the best action in that particular instantiation. The resulting final graph is the closest to the ground truth compared to the others.

For the other tests, we generated two DBNs using the equivalent algorithm for DBNs to NOTEARS, i.e., DYNOTEARS [45]. As we can see from Figure 3b,c, the result is only satisfying when we use a small threshold parameter, i.e., ϵ=0.01, which means that the structural equations of the causal graphs considered are shallow and therefore subjected to higher fluctuation given different datasets (Figure 3b). If we increase this parameter to ϵ=0.1, many connections will be erased, resulting in a sparse graph (Figure 3c). For the remaining tests, we used Direct LiNGAM [43], which is a method applied for learning causal structures from data without any further assumption, relying only on the statistical method known as independent component analysis, and VARLiNGAM [44], which corresponds to the previous algorithm for studying time series. We first derived the structural equations by studying only the features at one-time step through the LiNGAM method. The resulting graph is presented in Figure 3d. As we can see, we have some contradictory results since it seems that the reward influences the passenger’s position and, most importantly, the action, while it is well known that the action causes the reward. This error is shown in the next graph in Figure 3e, where, in this case, all the features of the memory dataset were used as different characteristics. Lastly, we generate the causal graph using VARLiNGAM, with threshold parameters for the relations between actual state features fixed at ϵ=0.01 and threshold for the next state features $\epsilon_{A}=0.1$. The obtained structure is shown in Figure 3f. The causal graph obtained presents the same problem with the causality of the reward, but, on the other hand, it can understand the relationship between the state features. In fact, it is the only graph, together with the initial one, to recognize the destination as a root node. Moreover, if we compare the graph obtained with the algorithm NOTEARS considering also the next time step state features, in Figure 3a and the one generated through VARLiNGAM, in Figure 3f, we can notice that the relations discovered are principally the same. For these reasons, we consider the structure obtained using the NOTEARS algorithm correct and the best in this set of different models.

After the creation of the DAG for the BN, it is possible to learn the probabilities using inference. To this end, the dataset must be discrete. The discretization of the features depends on the particular environment that is studied. In our case, we will focus on three RL benchmarks (Taxi, Cartpole and Mountaincar) that will be explained in detail in the next section. In a discrete environment, e.g., Taxi, every feature is ready for inference, while in a continuous one, e.g., Cartpole and Mountaincar, we have to find the best clusters for each characteristic using the K-means algorithm. Usually, for the discretization of RL environments, tile coding is applied, which consists in dividing the continuous space into different tiles and then for each point evaluating if it is in a particular tile (1) or not (0). In this way, it is possible to obtain a vector of 0s and 1s as a discrete representation of each value. This process, the One-Hot-Encoding, is completely independent of the values that will be used during the simulation. To avoid this, we used the K-means clustering algorithm thus we have a better differentiation between each cluster considering the information gathered during the training. More information about the creation of the clusters for each environment is given in the following section. After the discretization, we applied the Bayesian estimators’ method assuming a Dirichlet distribution prior to learning the probabilities of the BN.

Consequently, it is possible to forecast the next state values using the freshly created BN. In fact, by giving to the network the state information and the actual (counterfactual) action, the prediction for the future time step will be the output of the BN. Following this concept, we enunciate the definition of the actual (counterfactual) prediction.

**Definition** **7** **(Actual/Counterfactual Prediction).** *Given an initial vector of the state at a time t, $s_{t}$ and the actual (counterfactual) action $a_{t}$, the actual (counterfactual) prediction of the Bayesian Network $P_{act}$ ($P_{count}$) is defined as the state values with the maximum probability, given $s_{t}$ and $a_{t}$, i.e.:*

$$\left(P_{count}\right)P_{act}=\underset{s_{t+1}}{arg max}P\left(s_{t+1}|s_{t},a_{t}\right)$$

This definition will be used later for the description of how an explanation is generated.

**Figure 4:**
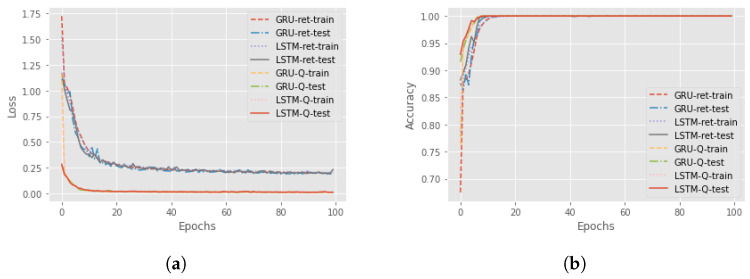
Comparison of losses and accuracy of RNNs with different cells and features for the Taxi problem. (**a**) Loss obtained during the training and test phase with different parameters. (**b**) Accuracy obtained during the training and test phase with different parameters.

Now, we can focus on distal predictions. The idea of distal actions was first presented in [8], where a distal action is one of the most significant actions that is enabled if the agent realizes a particular sequence of actions. However, there is also different influential distal information which is valuable for a more detailed explanation. An example could be the final return of the episode. The third and fourth components of the proposed methodology are RNNs for the actual and counterfactual distal predictions. The third component aims to predict the distal return and action/state following the correct policy, and the fourth component to forecast the distal values if, at a particular time step, an action different from the correct one is executed. For these networks, we compared the performance of Long-short term memory (LSTM) cells and Gated Recurrent Units (GRUs) to find the better option. The features involved in the training of the network were the state characteristics, the actions executed and the actual accumulated returns or the Q-values. Four different RNNs were created to understand the best choice: using LSTM cells or GRU and if it is better to consider the actual return or if it is sufficient to only use the Q-value. In Figure 4, there is a comparison between all four different networks in terms of loss and accuracy during the training and test session over 100 epochs, for the Taxi environment. It can be seen that the best choice is to use the GRU network with Q-value, but in general, there are no significant differences with the GRU network that used the return. For this reason, in an environment where we have the Q-value, it is better to use it as a feature, while, if the Q-function is not known, it is possible to accomplish the same result using the actual accumulated returns.

The main difference between the RNNs for the actual prediction and the counterfactual one is how the memory for their training is composed. The actual RNN data are created following the optimal policy and saving all the information of each step. Meanwhile, for the counterfactual, the agent will always choose the optimal action following the trained RL agent until a “why not” question is asked. In that case, the counterfactual action is chosen and saved in the same way as indicated before. The final return and the distal state/action are given by following the correct actions after the counterfactual one. In both cases, it is fundamental to pre-process data: for the actions, One-Hot-Encoding is applied, while for the return it was beneficial to standardize the values to reduce the time of the training and the loss.

**Figure 5:**
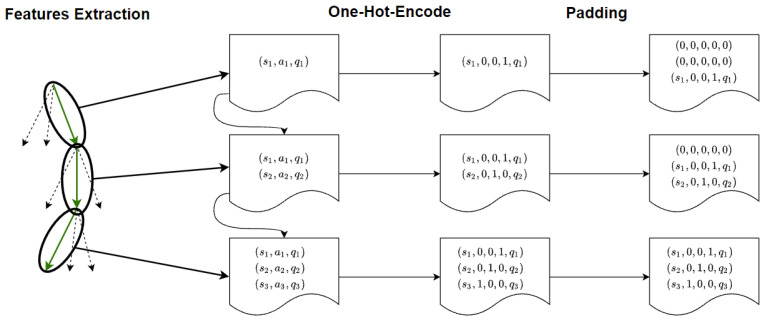
Creation of the dataset for the distal prediction.

Another important problem is that each data point is a time series of potentially different lengths. At the start of the episode, the saved values *v* are only the one of the initial time step, so $v\in R^{f\times 1}$ (where *f* represents the number of the features), while at the end, the saved data are composed of all the values of each previous step, i.e., if the episode length is *l* we have $v\in R^{f\times l}$. However, each data point must have the same dimensions. To this end, it is possible to evaluate the maximum length of the episodes *m*. Then, all the elements that have a lower dimension than *m* will be filled with vectors of 0. This technique (*Padding*) is a well known method used in message [46] or image recognition [47]. Figure 5 represents the pipeline of the creation of the dataset for the RNN training. Now, we introduce a definition of distal prediction that will be useful for creating the final explanation.

**Definition** **8** **(Distal Actual/Counterfactual Prediction).** *Given an initial vector of the state at a time t, $s_{t}$, the one-hot-encoded actual (counterfactual) action $a_{a}$ ($a_{c}$) and the corresponding Q-value $q_{t}$, the distal actual (counterfactual) prediction $d_{act}$ ($d_{count}$) is defined as the output of the actual (counterfactual) RNN given $s_{t},a_{t}$ and $q_{t}$ as input.*

All the information obtained from the previous components is fundamental for the explanation: the next state values prediction through the BN, the causal structure and the distal values obtained from the RNNs. However, the first step is to define what is the center of the explanation, i.e., the *central variable*.

**Definition** **9** **(Actual central variable/node for “why” question).** *Given the state $s_{t}$ and $P_{act}$, the vector of predicted values by the Bayesian Network at time step t, an actual central variable or node D in a Bayesian Network for the “why” question explanation is a node such that $s_{t}^{D}\neq P_{act}^{D}$, where $s_{t}^{D}$ and $P_{act}^{D}$ are, respectively, the D components of the state vector $s_{t}$ and the predicted vector $P_{act}$.*

After this introductory part, it is possible to talk about the explanation for “why” questions. The following definition shows in detail how to use the information obtained previously to create an explanation.

**Definition** **10** **(Explanation for “why” question).** *Given the state $s_{t}$ and an action $a_{t}$, the explanation for the “why” question is a tuple:*

$$(s_{t},a_{t},P_{act},d_{act},Pa\left(D\right),Ch\left(D\right),R\left(D\right)),$$

*where $P_{act}$ is the predicted values through the Bayesian Network, $d_{act}$ is the distal actual prediction through the actual RNN, Pa(D) is the parent set and Ch(D) the children set of the actual central node D and R(D) the reward node connected to D, which are the variables in the chain that are directly linked to the reward node.*

Principally, we give the user all the information obtained through the BN and RNN predictions, connecting all of them and analyzing the structure of the DAG.

Similar to the previous definitions, there are almost identical definitions for the counterfactual case.

**Definition** **11** **(Counterfactual central variable/node for “why not” question).** *Assume the state $s_{t}$ and let $P_{count}$ be the vector of the counterfactual predicted values by the Bayesian Network at a time step t. A central variable or node C in a Bayesian Network for a “why not” question explanation is the node such that $s_{t}^{C}\neq P_{count}^{C}$, where $s_{t}^{C}$ and $P_{count}^{C}$ are, respectively, the C components of the actual vector $s_{t}$ and the counterfactual predicted vector $P_{count}$.*

**Definition** **12** **(Explanation for “why not” question).** *Given the state $s_{t}$, an actual action $a_{a}$ and a counterfactual action $a_{c}$, the explanation for a “why not” question is a tuple:*

$$(s_{t},a_{a},a_{c},P_{act},d_{act},P_{count},d_{count},C,Pa\left(D\right),Ch\left(D\right),R\left(D\right)),$$

*where $P_{act},P_{count}$ are, respectively, the vectors of predicted actual and counterfactual values through the Bayesian Network, $d_{act},d_{count}$ are, respectively, the distal prediction through the actual and counterfactual RNNs, C is the counterfactual central variable/node, Pa(D) the parent set and Ch(D) the children set of the actual central node D and R(D) the reward node connected to D.*

For the explanation of the “why not” question, the main idea is the same as the actual one: give to the user all the predicted information, linking them with each other through the central variables and the structure of the BN. In this way, it is also possible to directly compare the results of actual and counterfactual forecasting. Therefore, human awareness of the correctness of the agent will increase. An interesting event is when we have more than one central variable. In these scenarios, we will compute the chain for all the nodes that satisfy the properties described above.

In the next section, all these definitions are practically used to clearly explain what the results of the model will be.

## 5. Results

For the evaluation of the quality of the methodologies in XRL, different metrics are used to compare how well one approach behaves with respect to others [48]. In our case, we considered an application level and, then, a human level, as proposed in [49]. In the first case, we focused our attention on the accuracy of the prediction made by the model. In this way, it is possible to directly confront the results achieved in other papers [8,9], obtained always in the same environments and using the same agents. In fact, if the forecasts of the agent’s actions and outcomes are wrong, all the resulting explanation will be completely erroneous. In the second case, applying a survey [49], it is possible for human users to judge the proposed explanations and give grades on a Likert scale base. Moreover, we also implemented other inquiries from [27]; thus, we are able to rank the different statements considering multiple qualities and properties.

Therefore, in the following sections, we reported the experiments and results in two parts: one for the computational analysis and the other for the human study.

### 5.1. Computational Evaluation

The new method is now tested in three different benchmarks environments from OpenAI Gym [50] (Taxi, Cartpole and Mountaincar), with distinct RL agents (SARSA, Policy Gradient and DQN). The specific details of each problem and examples of generated explanations are described in the following subsections. For the parameter choice, we refer to Appendix A. In particular, the parameters presented in Table A1 were selected using *optuna* 2.9.1 [51] after 100 trials, while the parameters of the RNNs, illustrated in Table A2, were fixed at the beginning of the test.

#### 5.1.1. Taxi

**Figure 6:**
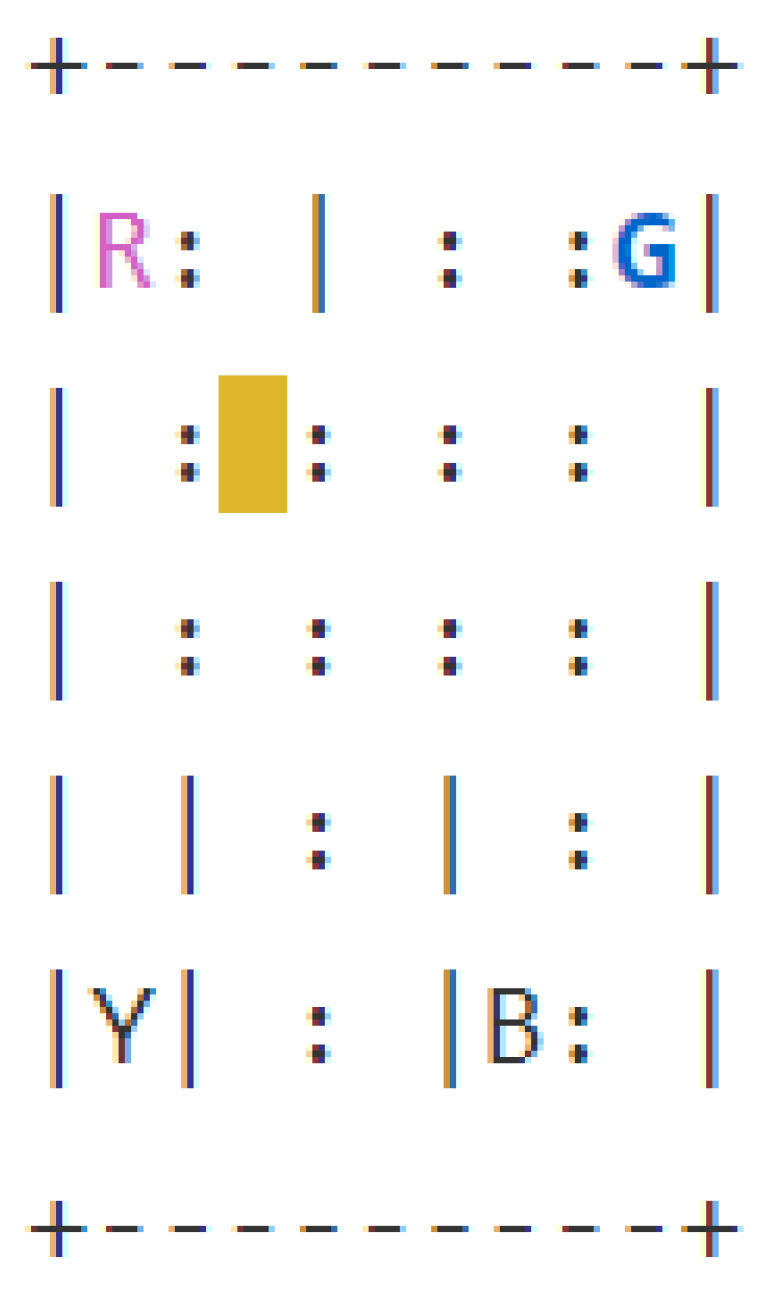
Rendering of the Taxi environment.

**Figure 7:**
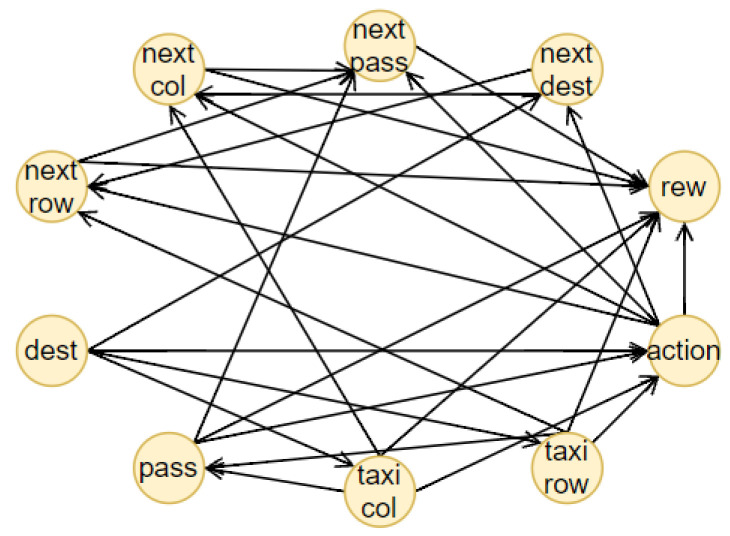
Structure of the BN for Taxi environment where each node represents a feature: “dest” is the destination, “pass” is the passenger position, “taxi col” is the taxi column, “taxi row” is the taxi row, “action” is the action, “rew” is the reward, “next dest” is the next value for the destination, “next pass” is the next value for the passenger position, “next col” is the next value for the taxi column and “next row” is the next value for the taxi row.

The Taxi environment [52], represented in Figure 6, is a well known benchmark environment for developing RL algorithms. The problem can be considered as a simplified real-life problem: at the beginning of the episode, the taxi starts at a random square in a 5×5 grid world and the passenger is at a random location. Then, the taxi driver has to drive to the passenger’s place, pick up the passenger, go to the passenger’s destination and drop off the traveller. The RL method used for the Taxi environment is Sarsa and Table A1 reports the parameters used for training. The structure of the BN, presented in Figure 7, is created using the NOTEARS algorithm (already implemented in the Python library *causalnex* [15]) with a thresholding parameter ϵ=0.5. For the analysis of the accuracy of the prediction, the agent completed 100 episodes and during each step the BN had to forecast the next action and next state values. The accuracy is reported in Table 1. After that, we calculated the AUROC to have a different measure of the quality of the prediction. In both cases, the results obtained are optimal.

**Figure 8:**
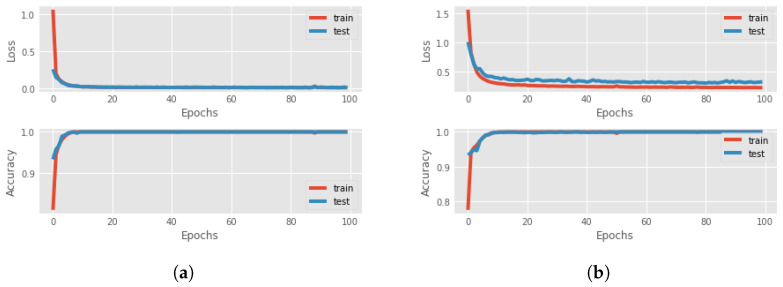
Results of the training and test of the RNNs for the distal prediction in the Taxi environment. (**a**) Loss and accuracy of the RNN for the distal actual prediction. (**b**) Loss and accuracy of the RNN for the distal counterfactual prediction.

The distal values that have to be predicted are the final return of the episode and the distal action. In the Taxi environment, the most significant action during the episode is picking up the passenger and dropping him off at the correct destination. So, all the actions before the right “pick up” have a distal action “pick up”, while, after that, all the other steps have a distal action “drop off”. For this reason, the dataset for the training of the RNNs is created by applying the idea above: fixing the distal action to “pick up” for all the time steps until the passenger is in the taxi and then it will be identified as “drop off”. The parameters of the RNNs are presented in Table A2 while Figure 8 shows the results of the training and test of the RNNs.

To understand how the explanation is produced, it is helpful to describe two examples: one for the “why” question and the other for the “why not” question. First, we consider how to answer the “why” question. Consider the actual state $s_{t}$ given by $s_{t}=\left(\mathrm{South}-\mathrm{East},\;\mathrm{South}-\mathrm{West},\;\mathrm{East},\;\mathrm{North}\right)$, where the first element is the destination, the second one is the passenger position and then the taxi position represented, respectively, in column and row. Consider the actual action $a_{a}=\mathrm{Go West}$ and the counterfactual $a_{c}=\mathrm{Go South}$. So the questions are: “why Go West?” and “why not Go South?”. The first step is evaluating the next state values through the BN. The results are the following: for the actual prediction, we obtain

$$P_{act}=\left(\mathrm{South}-\mathrm{East},\;\mathrm{South}-\mathrm{West},\;\mathrm{East}-\mathrm{Center},\;\mathrm{North}\right)$$

and for the counterfactual prediction

$$P_{count}=\left(\mathrm{South}-\mathrm{East},\mathrm{South}-\mathrm{West},\mathrm{East},\mathrm{North}-\mathrm{Center}\right).$$

Then, using the accumulated information during the episode, it is possible to predict the distal values through the trained RNNs. The actual value is

$$d_{act}=\left(\mathrm{Pick up},\;3.75\right)$$

while the counterfactual value is

$$d_{count}=\left(\mathrm{Pick up},\;0.73\right).$$

**Figure 9:**
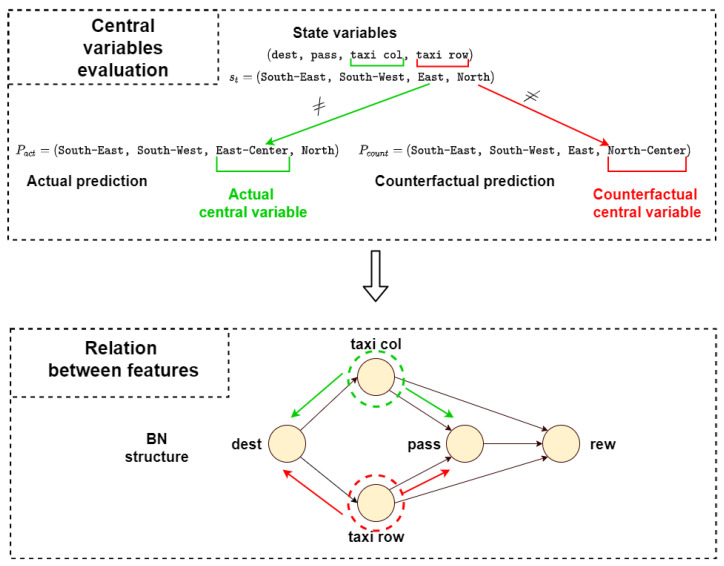
Evaluation of the central variables and estimation of the actual (green) and counterfactual (red) chains for the determination of the feature relations used in the explanation.

The last step before the creation of the explanation is finding the central variables in the structure of the BN, using Figure 7. The actual central variable is the taxi column (*D*) because $s_{t}^{D}=\mathrm{East}$ and $P_{act}^{D}=\mathrm{East}-\mathrm{Center}$, while the counterfactual central variable is the taxi row (*C*), since $s_{t}^{C}=\mathrm{North}$ and $P_{count}^{C}=\mathrm{North}-\mathrm{Center}$. Then, looking at the structure of the BN, it is possible to find automatically the actual and counterfactual chains composed of the parent and children set of the central variables and the nodes connected to the reward node in those chains. This process is summed up in Figure 9.

Then, the answer to the “why” question is:

“Since the Destination (Pa(D)) is South-East ($s_{t}^{Pa(D)}$), it is desirable to do action Go West ($a_{a}$) in order to change the Taxi column (*D*) from East ($s_{t}^{D}$) to East-Center ($P_{act}^{D}$) to influence the Passenger position (Ch(D)), because Passenger position and Taxi column (R(D)) are connected to the goal. Following this action, it will be possible to do Pick up the passenger and obtain a final return of 3.75 ($d_{act})$)”.

The answer to the “why not” question is instead:

“Since the Destination (Pa(D)) is South-East ($s_{t}^{Pa(D)}$), it is more desirable to do action Go West ($a_{a}$) in order to change the Taxi column (*D*) from East ($s_{t}^{D}$) to East-Center ($P_{act}^{D}$) instead of changing the Taxi row (*C*) from North ($s_{t}^{C}$) to North-Center ($P_{count}^{C}$) doing action Go South ($a_{a}$), to influence the Passenger position (Ch(D)), because Passenger position and Taxi column (R(D)) are connected to the goal. Following this action, it will be possible to do Pick up the passenger and obtain a final return of 3.75 ($d_{act})$, while following the counterfactual action will lead to Pick up the passenger and obtain a final return of 0.73 ($d_{count}$)”.

The structure of the explanation is created with the same idea for each environment: we start with the instantiation of the nodes in the parent set, then listing both actions (the actual and the counterfactual for the “why not” questions) and the predictions of the BN we introduce the central variable (comparing the actual instantiation with the predicted one) and finally, we describe the influences in the children and reward nodes. After this first sentence, there will be a distal explanation where the distal information will be reported.

#### 5.1.2. Cartpole

**Figure 10:**
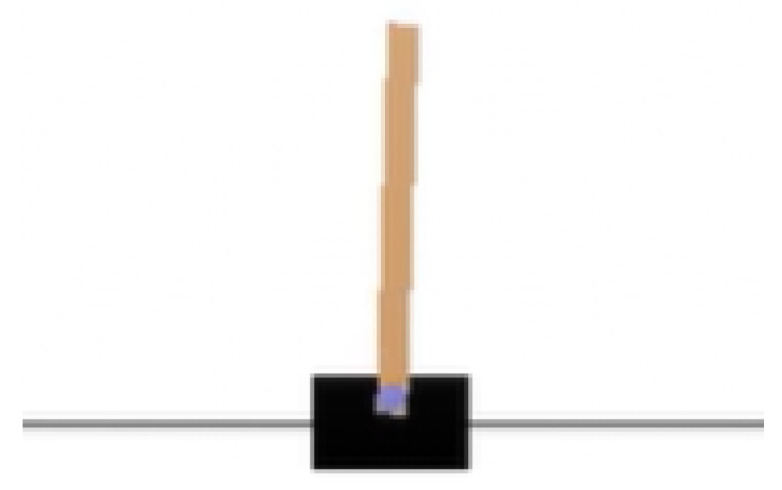
Rendering of the Cartpole environment.

The Cartpole environment, illustrated in Figure 10, is also a classical test problem used in RL. It was proposed and studied for the first time by Sutton and Barto [10]. A pole is attached by a joint to a cart, which moves along a one-dimensional track, without friction. The pole starts upright and the goal is to prevent it from falling over by increasing or reducing the cart’s velocity. Thus, the set of actions is composed of two possibilities: moving the cart to the left or the right. The state features are cart position, cart velocity, pole angle and velocity. The RL method used for this environment is Policy Gradient and Table A1 presents the parameters used.

**Figure 11:**
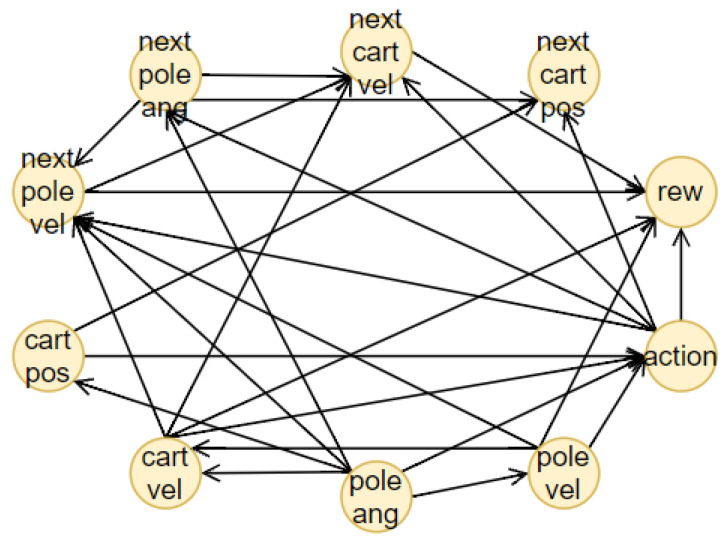
Structure of the BN for the Cartpole environment. The names of the nodes represent the following features: “cart pos” is the cart position, “cart vel” is the cart velocity, “pole ang” is the pole angle, “pole vel” is the pole velocity, “action” is the action, “rew” is the reward, “next cart pos” is the next value for the cart position, “next cart vel” is the next value for the cart velocity, “next pole ang” is the next pole angle, “next pole vel” is the next value for the pole velocity.

The structure of the BN, represented in Figure 11, is created using the NOTEARS algorithm with a thresholding parameter ϵ=0.8 (chosen after a comparison of the structure obtained). In this case, the environment is continuous, so all the features have to be discretized. The limit values for each bin are obtained using the K-means clustering algorithm, fixing the number of clusters to K=6, obtained from the elbow method. Table A3 presents the results.

The prediction accuracy is tested on 100 episodes and the results are shown in Table 2. These results are not as convincing as they were in the discrete environment (Taxi test problem). To increase the accuracy, it is possible to augment the number of discrete values. However, this solution has a weakness: using a large amount of different discretized values will expand the computation time of the predictions. For this reason, the number of clusters was fixed at six.

**Figure 12:**
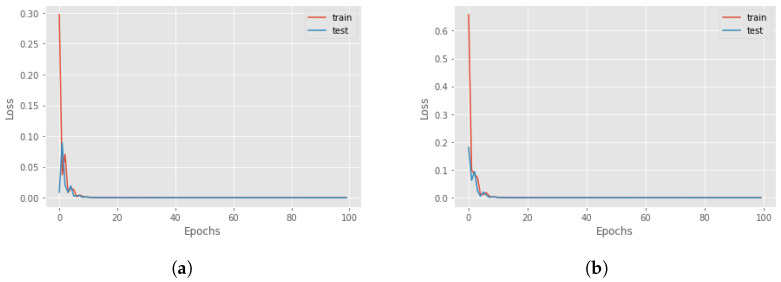
Results of the training and test of the RNNs for the distal prediction in the Cartpole environment. (**a**) Loss of the RNN for the distal direct prediction. (**b**) Loss of the RNN for the distal counterfactual prediction.

In this case, the distal value that has to be predicted is only the final return, since there are no other important characteristics that can be used for a better explanation. Another fundamental aspect to keep in mind is the lack of evaluation of the Q-value. Then, for this environment, we choose as distal information the actual return. As seen in Section 4, the prediction accuracy using the actual return is sufficiently good for our task. However, this was not the only problem for this environment. Since the length of each episode is 500 (in the worst case), this will lead to the creation of a large time-series dataset, that slows down the prediction and needs an enormous amount of free memory to store all the information. For this reason, after 100 steps we end all the episodes. The other parameters of the RNNs are presented in Table A2. The forecasting of these two RNNs is optimal in both phases, as can be seen in Figure 12.

Now, two examples of answers, in particular one for the “why” question and the other one for the “why not”, will be explained in detail. Consider the initial state values $s_{t}=(\mathrm{Center}-\mathrm{Left},\;\mathrm{Low Negative},\;\mathrm{Center}-\mathrm{Right},\;$Low Positive), where the first element is the cart position, then the cart velocity, the pole angle and lastly the pole velocity. The actual action $a_{a}=\mathrm{Go Left}$ and the counterfactual is $a_{c}=\mathrm{Go Right}$. The questions are “why Go Left?” and “why not Go Right?”. The results of the BN prediction are the following: the direct prediction is

$$P_{act}=\left(\mathrm{Center}-\mathrm{Left},\;\mathrm{Low Negative},\;\mathrm{Center}-\mathrm{Right},\;\mathrm{Positive}\right)$$

and the counterfactual is

$$P_{count}=\left(\mathrm{Center}-\mathrm{Left},\;\mathrm{Low Negative},\;\mathrm{Center}-\mathrm{Right},\;\mathrm{Low Negative}\right).$$

Then, using the time series information accumulated during the episode, it is possible to forecast the distal return: the actual

$$d_{act}=100.034$$

and the counterfactual

$$d_{count}=100.025.$$

The actual and counterfactual central variable is Pole Velocity (D,C) since $s_{t}^{D}=s_{t}^{C}=\mathrm{Low Positive}$, while the actual value is $P_{act}^{D}=\mathrm{Positive}$ and the counterfactual $P_{count}^{C}=\mathrm{Positive}$. After evaluating the corresponding parents and children sets through the structure of the BN, it is possible to create the answers. The answer to the “why” question is:

“Since Pole Angle (Pa(D)) is Center-Right ($s_{t}^{Pa(D)}$), it is desirable to do action Go Left ($a_{a}$) in order to change the Pole Velocity (*D*) from Low Positive ($s_{t}^{D}$) to Positive ($P_{act}^{D}$) to influence Cart Velocity (Ch(D)), because Pole Velocity and Cart Velocity (R(D)) are connected to the goal. Following this action, it will be possible to obtain a final return of 100.034 ($d_{act}$)”.

While the answer to the “why not” question is:

“Since Pole Angle (Pa(D)) is Center-Right ($s_{t}^{Pa(D)}$), it is more desirable to do action Go Left ($a_{a}$) in order to change the Pole Velocity (*D*) from Low Positive ($s_{t}^{D}$) to Positive ($P_{act}^{D}$) instead of changing the Pole Velocity (*C*) from Low Positive ($s_{t}^{C}$) to Low Negative ($P_{count}^{C}$) doing action Go Right ($a_{c}$), to influence Cart Velocity (Ch(D)), because Pole Velocity and Cart Velocity (R(D)) are connected to the goal. Following this action, it will be possible to obtain a final return of 100.034 ($d_{act}$), while following the counterfactual action will lead to a final return of 100.025 ($d_{count}$)”.

#### 5.1.3. Mountaincar

**Figure 13:**
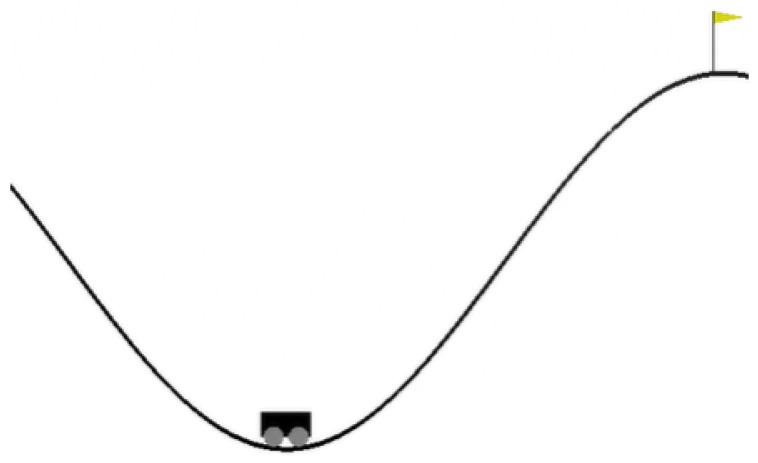
Rendering of the Mountaincar environment.

The Mountaincar environment, shown in Figure 13, is one of the most famous benchmark environments for testing RL algorithms. In this environment, a car starts at the bottom of a valley. Then, the agent can choose to accelerate to the left, right, or stop any acceleration. The goal of the car is to reach the top of the mountain on the right. However, it is impossible to go directly there, by exclusively accelerating to the right, because the car does not have enough power. For this reason, it has to create momentum to reach a higher position. In this case, the RL method applied is Deep-Q-Network [53] with parameters presented in Table A1.

**Figure 14:**
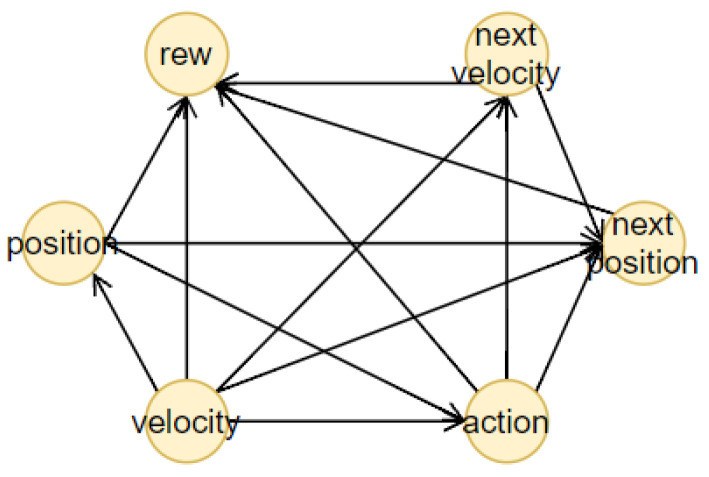
Structure of the BN for the Mountaincar environment. The names of the nodes represent the following features: “position” is the position of the car, “velocity” is the velocity of the car, “action” is the action, “next position” is the next position of the car, “next velocity” is the next velocity of the car and “rew” is the reward.

The structure of the BN—see Figure 14—is generated using the NOTEARS algorithm with the best thresholding parameter ϵ=0.8 obtained after a comparison of different results. We applied the K-means clustering algorithm and using the elbow method we chose the optimal values K=5 for the position and K=4 for the velocity. Table A4 reports the discretization values obtained. The prediction accuracy is tested in 100 episodes and the results are presented in Table 3. The AUROC value and the accuracy are high for each feature and the action prediction.

**Figure 15:**
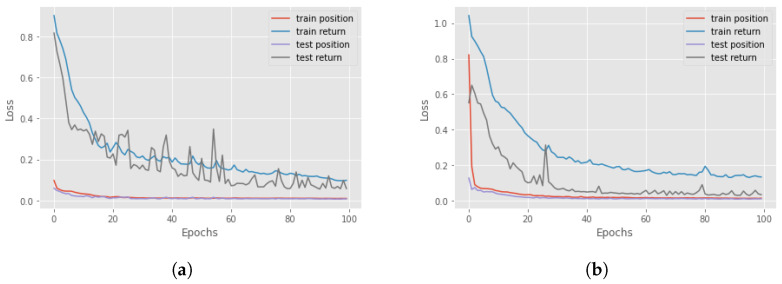
Results of the training and test of the RNNs for the distal prediction in the Mountaincar environment. (**a**) Loss of the RNN for the distal direct prediction. (**b**) Loss of the RNN for the distal counterfactual prediction.

The distal values for the Mountaincar problem are the final return and the highest position reached with that energy. In this way, it is possible to understand if the car can reach the top of the mountain or not. For the creation of the dataset, the highest position on the right is saved and all the past locations on that trajectory will have that as a distal position. The loss for both RNNs is represented in Figure 15 while the parameters are introduced in Table A2.

Also in this case, two examples will be used to clearly explain how to create the explanation. In the first example, the initial values $s_{t}=\left(\mathrm{Far}-\mathrm{Left},\;\mathrm{Low Negative}\right)$, where the first element is the position and the second is the velocity; the actual action $a_{a}=\mathrm{Go}\;\mathrm{Left}$ and the counterfactual is $a_{c}=\mathrm{Go}\;\mathrm{Right}$. The questions are “why Go Left?” and “why not Go Right?”. After that, one can predict the next state values through the BN. The results are the following: the direct prediction is

$$P_{act}=\left(\mathrm{Far}-\mathrm{Left},\;\mathrm{Negative}\right)$$

and the counterfactual

$$P_{count}=\left(\mathrm{Far}-\mathrm{Left},\;\mathrm{Low Positive}\right).$$

Then, using the time series information accumulated during the episode, it is possible to forecast the distal return and position: the actual

$$d_{act}=\left(1.28,154.40\right)$$

and the counterfactual

$$d_{count}=\left(1.04,154.13\right).$$

Then, the actual and counterfactual central variable is Velocity. Now, it is possible to create the answers through the structure of the BN. In this case, the answers will start differently because the parent set of the central variable is empty, so all the initial state values are considered. So the answer to the “why” question is:

“Since Position is Far-Left and the Velocity is Low Negative ($s_{t}$), it is desirable to do action Go Left ($a_{a}$) in order to change the Velocity (*D*) from Low Negative ($s_{t}^{D}$) to Negative ($P_{act}^{D}$) to influence Position (Ch(D)), because Velocity and Position (R(D)) are connected to the goal. Following this action, it will be possible to obtain a final return of 154.40 and a distal position of 1.28 ($d_{act}$)”.

While the answer to the “why not” question is:

“Since Position is Far-Left and the Velocity is Low Negative ($s_{t}$), it is more desirable to do action Go Left ($a_{a}$) in order to change the Velocity (*D*) from Low Negative ($s_{t}^{D}$) to Negative ($P_{act}^{D}$) instead of changing the Velocity (*C*) from Low Negative ($s_{t}^{C}$) to Low Positive ($P_{count}^{C}$) doing action Go Right ($a_{c}$), to influence the Velocity (Ch(D)) because Velocity and Position (R(D)) are connected to the goal. Following this action, it will be possible to obtain a final return of 154.40 and a distal position of 1.28 ($d_{act}$), while following the counterfactual action will lead to a final return of 154.13 and a distal position of 1.04 ($d_{count}$)”.

### 5.2. Human Evaluation Study

In order to evaluate the quality of the explanation generated using our methodology, we carried out a human study. In particular, we tested the hypothesis that our explanations lead to a better understanding of the RL agent strategies and an increase in trust in their choices.

**Figure 16:**
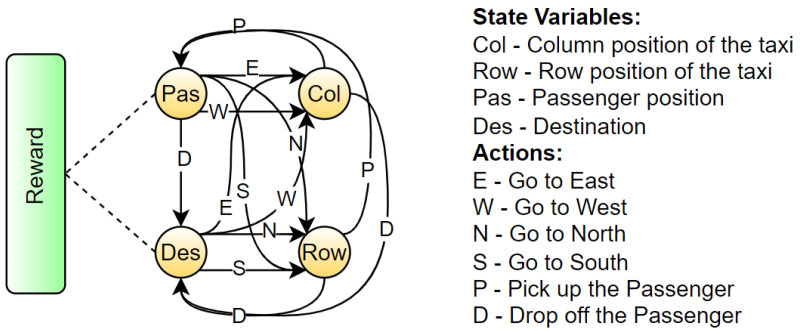
Action-Influence graph for the Taxi environment.

We decided to consider the Taxi environment, introduced in Section 5.1.1, in two different scenarios: we presented a “why” and a “why not” question with the corresponding explanations. For this study, we analyzed the distal explanations generated in [8,9] (C) for both the actual and counterfactual instance and the state relevant variables using template 1 of Khan et al. [27] (R) only for the “why” answer. Moreover, we also considered giving only the action chosen by the agent without any further explanation (N) to have a direct baseline. Therefore, our methodology (B) was compared with the three approaches in the actual scenario and two for the counterfactual. In detail, we present the following examples of the discussed explanations to increase clarity. For the sake of conciseness, we will consider only the situation where the taxi position is (North-Center, West), the passenger is in North-West and the destination is in South-West. For the relevant variables explanation (R), we used the original template with a small change: instead of counting the frequency of visiting a particular state, we considered the distance to the terminal state, since it was the relevant information in the Taxi environment. An example of (R) explanation is: “Why action Move North? Move North is the only action that is likely to take you to passenger=South-West, destination=South-West, taxi row=South, taxi column=West, in about 6 time steps, which is lower than any other action”. In the case of the causal explanations (C), first, we had to define an Action-Influence causal graph. Therefore, we considered the DAG obtained by applying our approach as a starting point and then, we developed the structure presented in Figure 16. For the choice of the optimal values, we manually set them knowing the task and how to reach the goal since the problem can be solved quickly by a human. Hence, considering the method described in Section 3.2, we obtain explanations like the following: “Why not action Move west? Because taxi row is not at the optimal number value North, it is more desirable do the action Move North to enable the action pick up as the goal is to have passenger and destination equal”. In the same way the explanations for the “why” questions were generated.

Lastly, for the generation of our explanation (B), we considered the associated BN, presented in Figure 7, together with its predictions and used the distal information computed from the RNNs to create the following statement: “Why not action Move west? Since the destination is South-West, it is more desirable to do action Move North in order to change the taxi row from North-Center to North instead of changing no values doing action Move west, to influence passenger because passenger, taxi row are connected to the goal. Following this action, it will be possible to do pick up and obtain a final return of 10, while following the counterfactual action will lead to pick up and obtain a final return of 7”. In the proposed scenario, we rounded the return forecast to the next integer value. The procedure is analogous for the “why” questions case, as presented in Section 5.1.1.

After the detailed description of the considered explanations, we focus now on the format of the questionnaire. The survey consists of three components: there is, first, an introduction to the environment and the agent task and then two sections, one for each particular question (“why” and a “why not”). In the latter cases, after a brief explanation of the context in which the agent takes action, we present an explanation at a time. Then, the participants can evaluate the quality of the explanations by answering questions introduced in [49] using a Likert scale: giving an integer evaluation between 1 (very bad) and 5 (very good). At the end of each question section, preference questions and specific true and false statements are given to collect more information useful for future works. For more information, we refer to Appendix B.

The participants of this survey were 30 students, self-selected at the University of Bundeswehr Munich and Camerino with engineering and scientific (mathematics, computer science and physics) backgrounds and the data were gathered in both physical and digital form. We can consider them experts since the task was a real-life application of a simple daily problem. Moreover, they received detailed clarifications about the environment where the explanations were introduced. In this way, we simulated an audience of domain experts which is one of the possible targets of XRL, together with non-experts in data science and AI [18,54,55].

**Figure 17:**
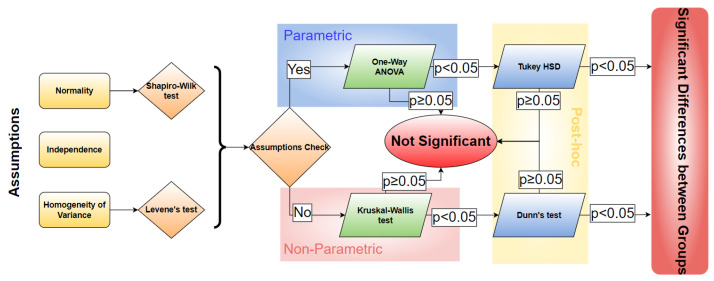
Pipeline of the statistical analysis of the data gathered in the human study.

For the analysis of the quality, we conducted a statistical study considering the pipeline presented in Figure 17. In detail, since the assumptions for the One-way ANOVA approach (Parametric method) applied in the previous studies [8,9] were not verified (for more detail, we refer readers to Appendix C), we had to use a non-parametric approach, i.e., Kruskal–Wallis test [56]. Subsequently, if the test is significant (*p*-value <0.05), i.e., there are significant differences between groups, we apply a post-hoc test to understand what the relations are between each kind of explanation. In this case, we considered Dunn’s test [57], which corresponds to the Tukey HSD test for the non-parametric situation. For the computation of the *p*-value for the latter test, we adopted the Bonferroni correction [58] to solve the multiple comparisons problem [59].

In the following, we analyze first the results for the “Why” explanations and then for the counterfactual question “Why not”.

#### 5.2.1. Evaluation of the “Why” Explanation

**Figure 18:**
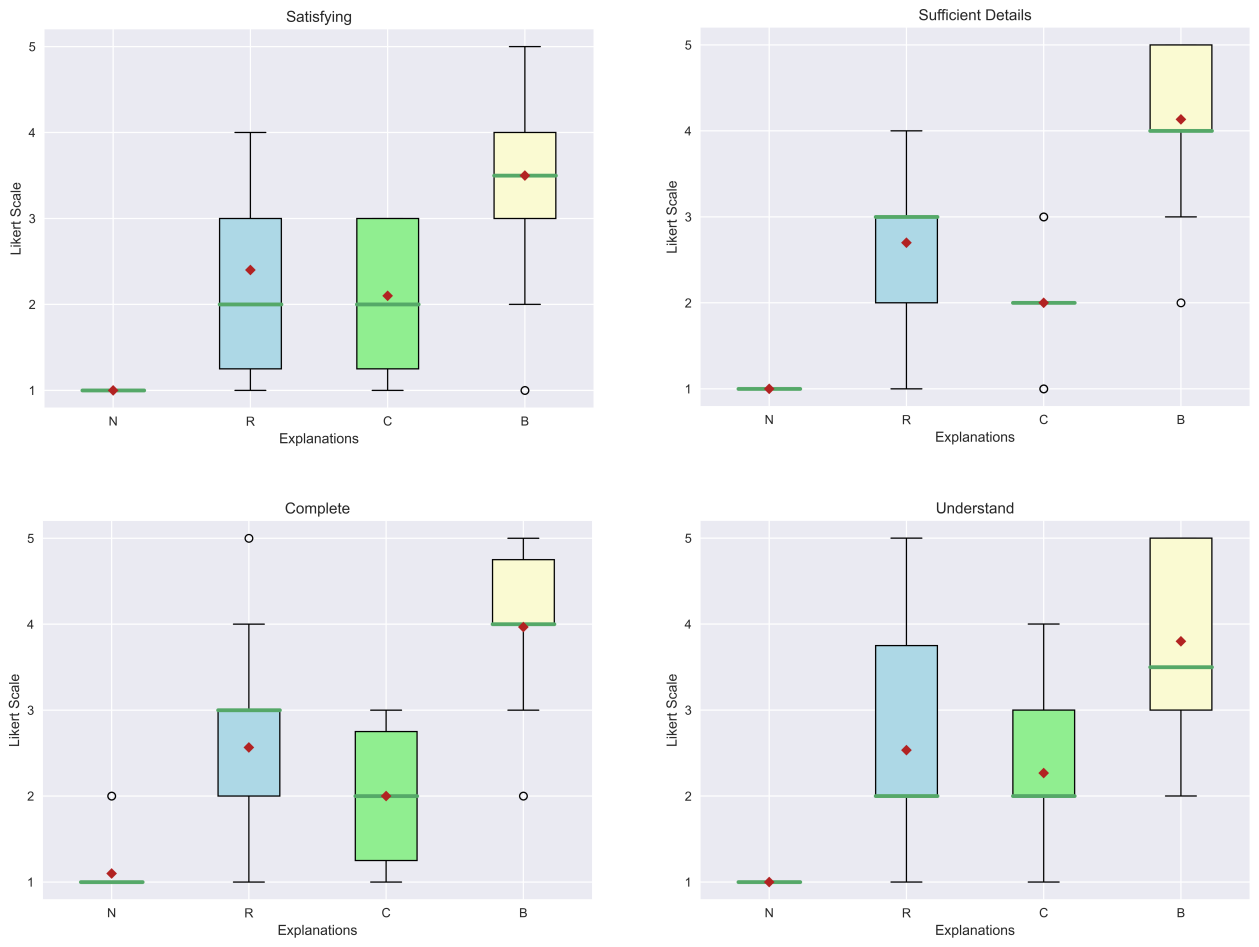
Box plot of the general qualities of the explanations for the “Why” question.

**Figure 19:**
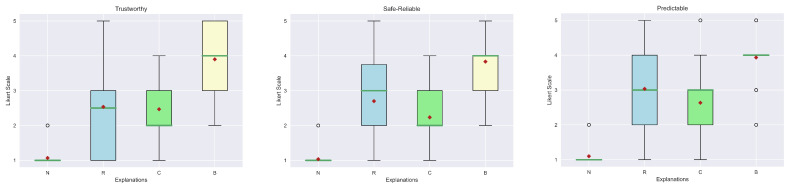
Box plot of the trust qualities of the explanations for the “Why” question.

For the evaluation of the explanations, we considered seven qualities from [49]: Understand, Satisfying, Sufficient Details, Complete, Trustworthy, Predictable and Safe-Reliable. The first four belong to the class of general qualities, while the latter are relatives of the trust area. The first step is to visualize the results obtained from the human evaluation in box plots. In particular, Figure 18 and Figure 19 show, respectively, the general qualities and the trust qualities. At first glance, it is possible to see how the median (green line) and mean (red diamond) of our explanation (B) are greater than the others. Another interesting aspect is that explanations (R) and (C) obtained similar scores. In fact, this kind of outcome was also reported in the study presented in [9]. In contrast, in our case, all the explanations are far better considered than the “No explanation” (N) approach, which can also be justified by the fact that in some contexts explanations may be not so relevant.

However, to have a clear view of the results obtained, it is fundamental to statistically analyze these data considering the methodology previously described. As shown in Table 4, the Kruskal–Wallis test reported a low *p*-value for each quality, confirming that there are differences between diverse explanation groups. Not only are the *p*-values below the usual threshold (0.05), but also the F-values are high (>2.69 critical value), i.e., the between-group variance is great or the within variance group is small or both of these conditions are verified. Therefore, a post-hoc test to individuate which groups differ from each other is necessary.

The results of Dunn’s test are shown in Table 5. As can be seen from the calculated *p*-values, all the explanations achieve better scores than “No explanation” (N). In particular, statistically, there are no differences in the relevant state variable explanations (R) and the Action-Influence one (C). Moreover, our answer is considered statistically different from both the previous explanations for all the qualities. The only value that is not significant is relative to the Predictable aspect when compared to (R).

**Figure 20:**
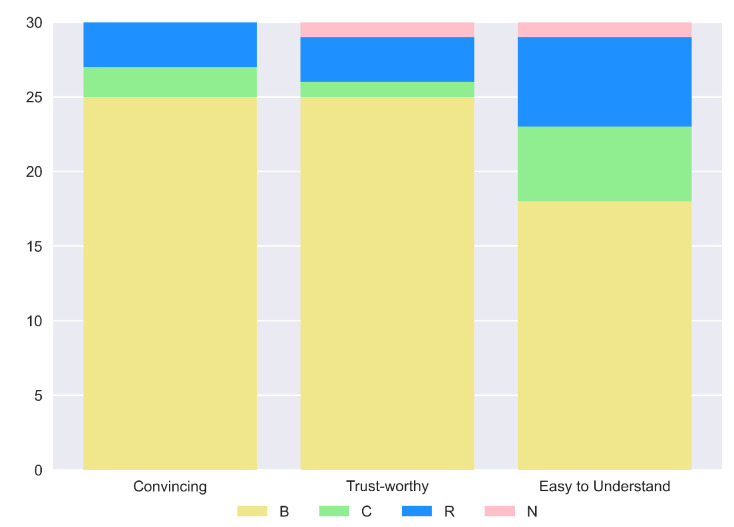
Bar chart of the preferences for actual explanations chosen by the participants for three different aspects.

**Figure 21:**
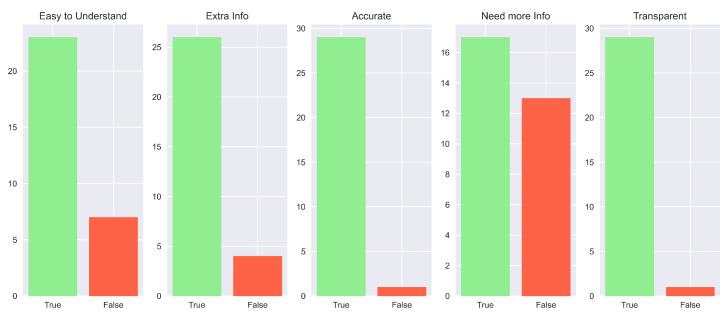
Bar charts representing the results of the True and False statements for different aspects of the actual explanations.

In general, these results are also supported by the overall consideration of the participants of the human study. In fact, in their preference questions, more than the majority of the study population answered that our explanation is the best in terms of “Convincing” (83.3%), “Trustworthy” (83.3%) and “Easy to Understand” (60%). Complete results are shown in Figure 20. Impressive results are found in the answers to the true and false statements, illustrated in Figure 21. In detail, a high number of participants agree that this kind of explanation is accurate (96.7%), transparent (96.7%) and provides more information (86.7%). An interesting result consists in the number of people who reported our explanation as “Easy to Understand” (76.7%). If we compare this number with the one obtained from the analysis of the preferences, we can notice that also people preferring other explanations as “the easiest to understand” consider ours good. The last remark is related to the question of the need for more information. In this case, there is a balanced result: 43.3% reported how our explanation should need more information; therefore, this request should be taken into account for possible future works.

#### 5.2.2. Evaluation of the “Why Not” Explanation

**Figure 22:**
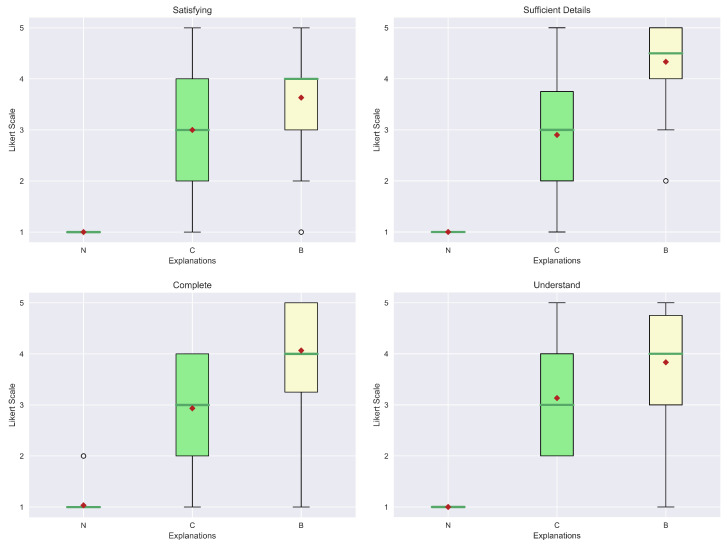
Box plot of the general qualities of the explanations for the “Why Not” question.

**Figure 23:**
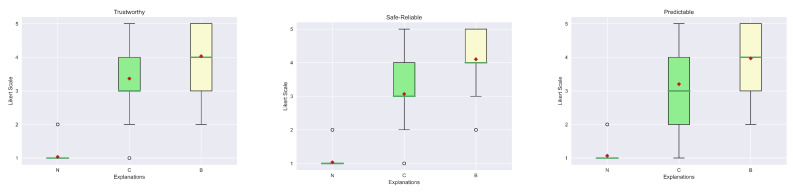
Box plot of the trust qualities of the explanations for the “Why not” question.

As described in the previous section, also during the analysis of the data gathered from the human study for the counterfactual explanations, we first visualized them in box plots, to understand the possible relations. The general qualities (Understand, Satisfying, Sufficient Details, Complete) and the ones related to trust (Trustworthy, Predictable and Safe-Reliable) are presented in Figure 22 and Figure 23, respectively.Already it is possible to see some small differences between the two explanations in terms of medians and means for each aspect. However, in this case, the representations of the 25th and 75th percentile testify that a complete statistical analysis has to be performed since these depictions could not be significantly used to deduce a major difference between groups.

Therefore, the Kruskal–Wallis test was executed obtaining the results presented in Table 6. Also in this case, a significant difference between groups is found since we obtain small *p*-values and great F-values for each quality. A remark can be made: if we compare these results with the ones obtained after the same test for the actual explanations, we can observe how the previously reported values are more significant, i.e., smaller for the *p*-values and greater for F-values, having then a slight difference in the comparisons of the groups. Thus, we expected to have less significant differences between our explanation and the one generated through the Action-Influence graphs.

This hypothesis is verified by using the multiple comparisons of Dunn’s test. As previously specified, all the *p*-values reported in Table 7 are corrected using the Bonferroni adjustment. The results presented highlight how, also in this situation, both the explanations overwhelm the no explanation case. However, interesting observations can be deduced from the comparison between our proposed answer and the one generated by the Action-Influence graph. As expected from the consideration of the previously performed Kruskal–Wallis test, significant differences are spotted only in three cases; in particular for Sufficient Details (0.001), Complete (0.015) and Safe-Reliable (0.020). In all the other aspects, a *p*-value lower than the significant threshold of 0.05 was not achieved. Therefore, we cannot deduce the presence of major differences in the compared explanations for these traits. Moreover, it is interesting to focus on Sufficient Details, since this is the quality that attains the lowest *p*-value. Substantially, this means that our explanation presents a higher number of details which can be useful for human users to better understand the choice of the agent.

**Figure 24:**
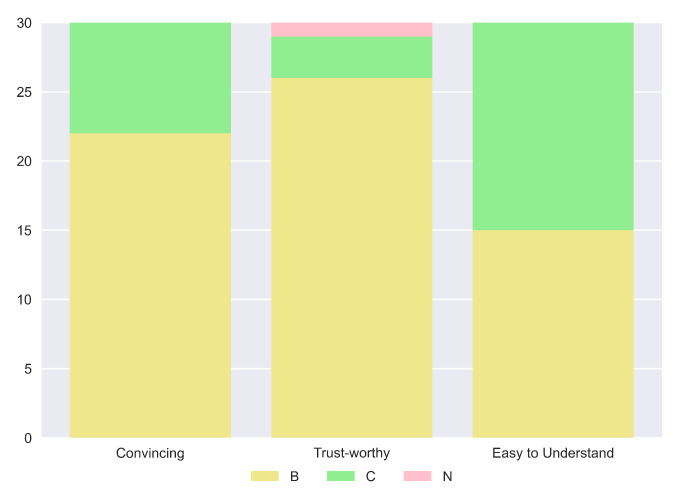
Bar chart of the preferences for counterfactual explanations chosen by the participants for three different aspects.

**Figure 25:**
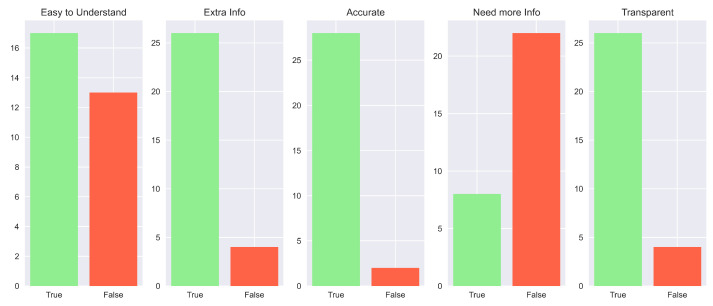
Bar charts representing the results of the True and False statements for different aspects of the counterfactual explanations.

However, increasing the amount of information given can lead to a trade-off in comprehensibility. If we look at the results in terms of preferences for the easiest explanation to understand (Figure 24), it is possible to see how half of the participants chose (B) and the other half (C). Moreover, from the true and false statement data shown in Figure 25, we can see how the 43.3% of the human study population considers our explanation not easy to understand. Hence, it is very important to balance the amount of information and the clarity of the final answer produced. On the other hand, also in this case, a high percentage of the participants voted the outcome of our method as the best in terms of being Convincing (73.3%) and Trustworthy (86.7%). Consequently, despite not having a clear significant difference in some qualities of the explanations, we can recognize our description as better than the one proposed in [8].

The last remarks concerning Figure 25 are related to the answers given for the necessity of more information. In fact, while for the other aspects we obtain results that are comparable to the “Why” scenario, e.g., gives extra information (86.7%), is accurate (93.3%) and transparent (86.7%), in the “Need more Info” statement there is an opposite result: 76.7% of participants agreed that our explanation does not need other details. This means that the counterfactual answer gives enough data to increase the comprehensibility of the agent’s actions.

## 6. Discussion

We tested the prediction accuracy of each model on 100 episodes after the training and the results can be seen in Table 8. The table shows the dimensions of each environment, where the first number represents the amount of the state features and the second one the possible actions. The results for the Mountaincar and Taxi environment are much better than those for the Cartpole experiment. This low accuracy is due to the difficulty of identifying the correct velocity of the pole and the cart. The number of clusters used for the discretization needs to be increased at the expense of a higher computational time to obtain better feature predictions. In general, our method seems to perform well in a discrete environment while losing some accuracy in the continuous case due to discretization.

**Figure 26:**
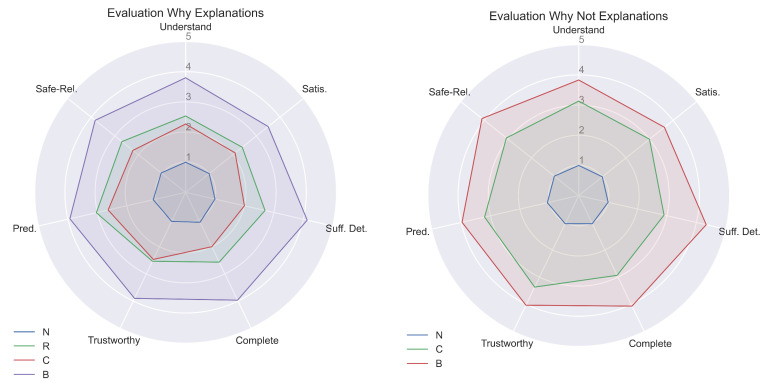
Radar Plot with the means of the scores achieved for each quality in both the “Why” and “Why not” explanations.

Focusing now on the explanation created, it is possible to understand the improvements obtained. Statistically relevant differences are found during the human study for both the actual and counterfactual explanations. In particular, in the first case, our approach scored way higher grades compared to the baseline explanations for all the aspects. Alternatively, for the counterfactual scenario, the number of statistically significant increments in quality is lower. However clear differences can be seen by analyzing both the explanations in detail. In fact, the answer to the counterfactual question in the Starcraft II environment of [8], presented in Section 3, can be compared to the ones obtained in our experiments, which are described in Section 5.1.1. From this comparison, we can see that our explanations have more detailed information because of the predicted future state values obtained from the BN. Using all the predicted subsequent state values provides more transparency to what will happen and will be trusted more by the user, as verified by the answers in the human study. Another interesting observation can be made by analyzing the use of distal information in both the explanation in counterfactual cases. In Madumal et al.’s example [8,9], it is used to directly understand the goal of the previous actions, while in our case, we use them to confront the outcome of the actual action to the counterfactual one. In fact, the possibility to compare the predictions in the case of counterfactual actions can reassure one that the actual action was the correct one. For this reason, using two different RNNs trained for specific distal prediction for direct and counterfactual cases is fundamental for a convincing explanation. Overall, we can state that humans value our answers in a superior way compared to the other baselines, as confirmed in the radar plot of Figure 26 showing the means of the scores achieved for both the actual and counterfactual cases. Therefore, with our approach, we can generate higher quality explanations and also increase the trust in the agent’s choices.

Lastly, we recall that the final task of the explainable approaches is to be adopted in real-world situations. This means that these methodologies have to consider complicated problems and very complex models, which usually present millions of parameters. For this reason, it is fundamental to highlight approaches such as the one proposed here, which can be applied in different environments and general agents. Moreover, introducing this explainable component does not bring any changes or requests in the design of the RL algorithm.

## 7. Conclusions and Future Work

This paper introduces improvements in the existing explanation method of Madumal et al. [8,9] for model-free RL agents that can create answers to “why” and “why not” questions. This model can predict the future state values and the distal information, in both cases (actual and counterfactual). These predictions are obtained by using a BN created from the data saved during the training phase of the RL agent. Specifically, the structure is the output of the NOTEARS algorithm and the probabilities are learnt using the Bayesian Estimator method. The distal values are the outcome of two different Recurrent Neural Networks composed of GRUs. The explanation is finally generated using the predicted information and the BN structure for the causal relations of the features.

This approach is then computationally evaluated using three RL benchmark environments. The results showcase the quality of the method for the discrete environment, although it has some weaknesses in continuous scenarios. In fact, in these cases, a discretization has to be performed and this leads to a decrease in the prediction accuracy. After that, a human study is proposed to obtain a clear view of how users can perceive our explanation compared to the other baseline. The analysis of the participants’ answers proves the quality of our answers and emphasizes a statistically significant difference between the different approaches for the actual scenario. Moreover, although we did not find many significant results for the counterfactual questions, we can conclude that our description is better perceived since it achieved higher means and medians.

The highlights of the proposed model are the ability to evaluate the future state values with high accuracy and the possibility to compare the distal prediction for the actual and counterfactual actions. These solutions allow the user to better understand why the machine is doing that particular action and give the people more trust.

Future work will involve the solution of continuous scenario problems and an application to a larger environment to test the reliability of this model in complex cases. For example, it could be opportune to prove this approach in the Starcraft II scenario. Then, it will be possible to compare the results obtained by the improved method and the past works of Madumal et al. [8,9]. Moreover, as depicted from the human study, it could be useful to increase the amount of information given in the explanation, introducing other fundamental relationships, e.g., connections between features in different time steps. In this case, it is important to recall how also this could compromise the readiness of the explanation. Therefore, studying an answer that provides a large number of details can help us to understand what is the limit that has to not be surpassed. Another interesting future study could be related to the choice of distal information. In our case, an expert in the domain chose the distal information. Thus, to make this model less human-dependent, an analysis of the data has to be performed to automatically find these data.

## Figures and Tables

**Table 1:** AUROC and accuracy for the prediction of each future feature in the Taxi environment.

Parameter	Action	Column	Row	Passenger	Destination
AUROC	0.857	0.996	1.000	1.000	1.000
Accuracy	0.994	1.000	1.000	1.000	1.000

**Table 2:** AUROC and accuracy for the prediction of each future feature in the Cartpole environment.

Parameter	Action	Cart Position	Cart Velocity	Pole Angle	Pole Velocity
AUROC	0.804	0.997	0.927	0.983	0.946
Accuracy	0.767	0.988	0.582	0.917	0.652

**Table 3:** AUROC and accuracy for the prediction of each future feature in the Mountaincar environment.

Parameter	Action	Position	Velocity
AUROC	0.948	0.994	0.990
Accuracy	0.859	0.952	0.980

**Table 4:** Table of the F-values and *p*-values obtained in the Kruskal–Wallis test for the actual explanations.

	F-Value	*p*-Value
Understand	72.960691	9.910749 $\times \;10^{-16}$
Satisfying	62.098886	2.092725 $\times \;10^{-13}$
Sufficient Details	88.564855	4.454306 $\times \;10^{-19}$
Complete	74.628244	4.352924 $\times \;10^{-16}$
Trustworthy	65.861863	3.280842 $\times \;10^{-14}$
Predictable	72.218146	1.429504 $\times \;10^{-15}$
Safe-Reliable	72.177046	1.458781 $\times \;10^{-15}$

**Table 5:** Table of the *p*-values obtained from Dunn’s test for the comparison of the actual explanations.

	R—N	C—N	B—N	C—R	B—R	B—C
Understand	<0.001	<0.001	<0.001	1.000	0.004	<0.001
Satisfying	<0.001	<0.001	<0.001	1.000	0.020	0.001
Sufficient Details	<0.001	0.001	<0.001	0.327	0.002	<0.001
Complete	<0.001	0.008	<0.001	0.746	0.001	<0.001
Trustworthy	<0.001	<0.001	<0.001	1.000	0.001	0.002
Predictable	<0.001	<0.001	<0.001	1.000	0.057	0.001
Safe-Reliable	<0.001	<0.001	<0.001	1.000	0.009	<0.001

**Table 6:** Table of the *p*-values obtained from Kruskal-Wallis’s test for the comparison of the counterfactual explanations.

	F-Value	*p*-Value
Understand	64.481313	9.955449 $\times \;10^{-15}$
Satisfying	61.631290	4.139381 $\times \;10^{-14}$
Sufficient Details	71.454554	3.046777 $\times \;10^{-16}$
Complete	64.447933	1.012300 $\times \;10^{-14}$
Trustworthy	63.839649	1.372134 $\times \;10^{-14}$
Predictable	60.428756	7.552008 $\times \;10^{-14}$
Safe-Reliable	67.743373	1.948558 $\times \;10^{-15}$

**Table 7:** Table of the *p*-values obtained from Dunn’s test for the comparison of the counterfactual explanations.

	C—N	B—N	B—C
Understand	<0.001	<0.001	0.226
Satisfying	<0.001	<0.001	0.376
Sufficient Details	<0.001	<0.001	0.001
Complete	<0.001	<0.001	0.015
Trustworthy	<0.001	<0.001	0.240
Predictable	<0.001	<0.001	0.192
Safe-Reliable	<0.001	<0.001	0.020

**Table 8:** Comparison of the accuracy (%) in prediction obtained with different methods: LR is the Linear Regression, DT is the Decision Tree, MLP is the Multi-Layer Perceptron, DP is the Decision Policy Tree, $DP_{n}$ is the Decision Policy Tree with a fixed depth and BN is the Bayesian Network.

Env-RL	Size	Past Works [8,9]					Ours
		LR	DT	MLP	DP	${\mathrm{DP}}_{n}$	BN
Cartpole-PG	4/2	83.8	81.6	86.0	96.83	**97.10**	78.13
MountainCar-DQN	2/3	69.7	57.8	69.6	88.66	86.75	**93.03**
Taxi-SARSA	4/6	68.2	74.2	67.9	82.44	86.19	**99.88**

**Figure A1:**
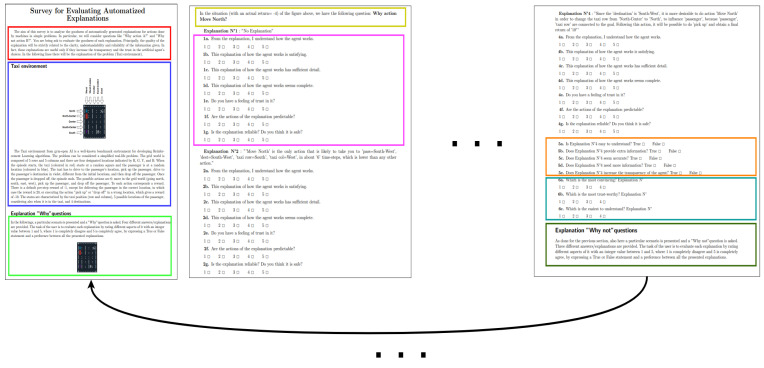
Structure of the survey.

**Figure A2:**
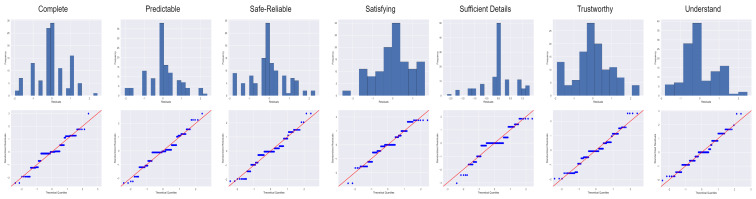
Histograms of the distribution of the residuals and Q-Q plots for each quality of the “Why” explanations.

**Figure A3:**
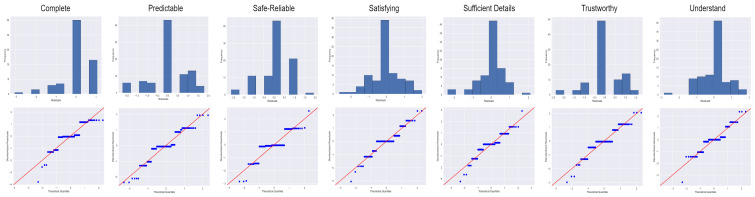
Histograms of the distribution of the residuals and Q-Q plots for each quality of the “Why not” explanations.

## Data Availability

The data presented in this study are available on request from the corresponding author.

## Abbreviations

The following abbreviations are used in this manuscript:

ANN	Artificial Neural Network
AUROC	Area Under the Receiver Operating Characteristic
BN	Bayesian Network
DAG	Directed Acyclic Graph
DBN	Dynamic Bayesian Network
DNN	Deep Neural Network
DQN	Deep Q-Network
GRU	Gated Recurrent Unit
LSTM	Long Short-Term Memory
MDP	Markov Decision Process
ML	Machine Learning
RL	Reinforcement Learning
RNN	Recurrent Neural Network
XRL	Explainable Reinforcement Learning

## Appendix A

In the following, the table relative to the parameter used for the training of RL algorithms (Table A1), training of the RNNs (Table A2) and the discretization bins for Cartpole (Table A3) and Mountaincar (Table A4) environment are presented.

**Table A1:** Parameter for the training of the different RL agents: α is the learning rate, γ is the discounting factor, ϵ is the value for the ϵ-greedy method, Δϵ is the decay (multiplicative for DQN and subtractive for PG), $\epsilon_{m}$ is the minimum value reachable by ϵ, $ep_{t}$ is the number of the episodes for the training, Ret is the average return after the training, $N_{h}$ is the number of hidden nodes and topology of the network, $f_{h}$ is the activation function. When a parameter was not considered, we represented this situation with the symbol ×.

RL	α	γ	ϵ	Δϵ	$\epsilon_{m}$	${\mathrm{ep}}_{t}$	Ret	$N_{h}$	$f_{h}$
Sarsa	0.1	0.95	0.4	0.001	0.01	5000	7.904	×	×
DQN	0.01	0.95	1	0.995	0.01	200	150.03	20.25	ReLU
PG	0.2	0.99	×	×	×	500	498.07	10	tanh

**Table A2:** Parameter for the training of the RNNs for the actual and counterfactual prediction in different environments: $N_{GRU}$ is the number of GRU, $f_{h}$ is the activation function used, $ep_{t}$ is the number of episodes for the dataset creation, $l_{M}$ is the maximum length of the episodes, bs is the batch size, epoc is the number of epochs, opt is the optimizer used and loss is the loss function (where MSE is Mean Square Error and SCC is Sparse Categorical Crossentropy).

Env	$N_{\mathrm{GRU}}$	$f_{h}$	${\mathrm{ep}}_{t}$	$l_{M}$	bs	Epoc	Opt	Loss
Taxi	50	tanh	1000	×	64	100	Adam	MSE/SCC
Cartpole	50	tanh	200	100	4096	100	Adam	MSE
Mountaincar	50	tanh	100	200	1024	100	Adam	MSE

**Table A3:** Discretized values for each feature in the Cartpole environment: these intervals are obtained using the K-means clustering method with K=6.

Features	Bin 1	Bin 2	Bin 3	Bin 4	Bin 5	Bin 6
Cart position	[−4.8, −0.85]	[−0.85, −0.20]	[−0.20, 0.30]	[0.30, 0.80]	[0.80, 1.30]	[1.30, 4.8]
Cart velocity	(−∞, −0.70]	[−0.70, −0.25]	[−0.25, 0.10]	[0.10, 0.45]	[0.45, 1.00]	[1.00, ∞)
Pole angle	(−0.418, −0.10]	[−0.10, −0.05]	[−0.05, 0.00]	[0.00, 0.04]	[0.04, 0.09]	[0.09, 0.418)
Pole velocity	(−∞, −0.42]	[−0.42, −0.20]	[−0.20, 0.00]	[0.00, 0.20]	[0.20, 0.42]	[0.42, ∞)

**Table A4:** Discretized values for each feature in the Mountaincar environment: these intervals are obtained using the K-means clustering method with K=5 for the position and K=4 for the velocity. For this reasons, we used the symbol × to represent the absence of the fifth bin.

Features	Bin 1	Bin 2	Bin 3	Bin 4	Bin 5
Position	[−1.2, −1]	[−1, −0.6]	[−0.6, −0.40]	[−0.40, 0.50]	[0.50, 0.60]
Velocity	[−0.07, −0.02]	[−0.02, 0]	[0, 0.02]	[0.02, 0.07]	×

## Appendix B

In this appendix, we illustrate Figure A1 that reports the survey in its main components. This questionnaire is divided into three main parts: an introduction giving the motivation of the study and the description of the problem, then the explanations for the “Why” question and lastly for the “Why not” case. Now, we specify the details relative to all the sections. The first main segment is composed of two blocks: the red one and the blue one. The red block presents all the aspects fundamental to understanding the aim of this study. Moreover, we give an outline of what will be the structure of the survey and the task of the participants. After that, in the blue rectangle, there is an accurate description of the Taxi environment, together with a picture representing the different names for each row and column. After the preliminary part, we can now move to the questionnaire, starting with the analysis of the “Why” questions section. In this case, the particular instance is presented in the light green block and the question with the action chosen by the agent is conferred in the yellow rectangle. Then, each explanation is firstly introduced and subsequently evaluated using a Likert scale between 1 (worst) and 5 (best), considering the questions and characteristics of [49]. This evaluation of the explanations’ qualities is repeated for each methodology and, in the end, other investigations are performed. In fact, following the ideas of the estimations done in [27], we used true and false statements (in the orange block) and preference inquiries (in the turquoise rectangle) to have a general overview of these methods. Lastly, the “Why not” questions section is introduced (in dark green). In this case, we reported the same structure as the previous part, analyzing each explanation’s qualities and in the end considering the best overall methodologies.

## Appendix C

In this appendix, we describe the detail for the check of the assumptions in statistical analysis for the comparisons of explanations in terms of different qualities. In particular, we consider, for each quality, as done previously, the two cases of actual (“Why”) and the counterfactual (“Why not”) explanations.

The first assumption that has to be checked is the independence of the data gathered. This means that all the groups are mutually exclusive and there should be no repeated measures, i.e., not collected through time. In general, this assumption is verified through a correct design of the study. Since in our case we tested different users at the same time, we have that the data collected are independently sampled.

The second assumption that has to be checked is the normality of the distributions of the residuals (experimental errors). In this case, the test applied is the Shapiro-Wilk test [60]. The results are represented in Table A5 for the actual explanations and in Table A6 for the counterfactual ones. We can notice that in both scenarios, the *p*-values are very low (≪0.05), meaning that we have to reject the hypothesis of the normality in the distribution of the residuals.

**Table A5:** Shapiro-Wilk test results for “Why” explanations.

	W-Test Statistic	*p*-Value
Understand	0.964634	3.036418 $\times \;10^{-3}$
Satisfying	0.950935	2.532099 $\times \;10^{-4}$
Sufficient Details	0.900190	2.006698 $\times \;10^{-7}$
Complete	0.939832	4.170681 $\times \;10^{-5}$
Trustworthy	0.955970	6.087143 $\times \;10^{-4}$
Predictable	0.947608	1.448555 $\times \;10^{-4}$
Safe-Reliable	0.951332	2.709522 $\times \;10^{-4}$

**Table A6:** Shapiro-Wilk test results for “Why not” explanations.

	W-Test Statistic	*p*-Value
Understand	0.904043	6.166087 $\times \;10^{-6}$
Satisfying	0.928571	9.938866 $\times \;10^{-5}$
Sufficient Details	0.913700	1.754128 $\times \;10^{-5}$
Complete	0.846812	3.270933 $\times \;10^{-8}$
Trustworthy	0.921450	4.244336 $\times \;10^{-5}$
Predictable	0.921005	4.029907 $\times \;10^{-5}$
Safe-Reliable	0.836157	1.415187 $\times \;10^{-8}$

This test can be validated also using particular plots, e.g., the Q-Q plots (Quantile-Quantile plots). As reported previously, also in this case we split the plots into actual (Figure A2) and counterfactual (Figure A3) instances. In particular, we represented in the first line the histograms of the distribution of the residuals and in the second line, the Q-Q plots are reported. In the histograms, the distributions do not look normal since there are still gaps between different values created by the characteristic of the scale used: this is a usual problem when an ordinal scale, i.e., the Likert scale, is used. This evidence is also confirmed in the Q-Q plots. As the standardized residuals do not lie around the y=x line, it suggests that the residuals are not normally distributed, therefore we can conclude that the assumption of normality is not satisfied.

The last assumption that we need to check is the Homogeneity of variances when the data is not drawn from a normal distribution. To this end, we apply Levene’s test for the homogeneity [61]. As done previously, we reported the W-test statistic and the *p*-value calculated for each quality in Table A7 for the explanation of the “Why” question and Table A8 for the “Why not” scenario. The results of the calculations prove that we must reject the hypothesis of homogeneity as well since the *p*-values are always below the threshold of 0.05 and also the W-test statistic confirms it since it is less than 152 (n=30). Therefore, the assumptions for considering a parametric test, e.g., one-way ANOVA and consequently, an associated post-hoc test, e.g., Tukey’s Honest Significant Difference (HSD), do not hold. Hence, the other possible choice is to consider a non-parametric test like the Kruskal–Wallis test and the post-hoc Dunn’s test.

**Table A7:** Levene’s test results for “Why” explanations.

	W-Test Statistic	*p*-Value
Understand	16.086792	1.135684 $\times \;10^{-6}$
Satisfying	25.951825	1.448917 $\times \;10^{-9}$
Sufficient Details	19.252912	1.194562 $\times \;10^{-7}$
Complete	14.500000	3.674218 $\times \;10^{-6}$
Trustworthy	28.195339	3.634358 $\times \;10^{-10}$
Predictable	12.537488	1.642332 $\times \;10^{-5}$
Safe-Reliable	18.283280	2.351775 $\times \;10^{-7}$

**Table A8:** Levene’s test results for “Why not” explanations.

	W-Test Statistic	*p*-Value
Understand	18.520000	1.991366 $\times \;10^{-7}$
Satisfying	20.056387	6.868431 $\times \;10^{-8}$
Sufficient Details	22.037906	1.806576 $\times \;10^{-8}$
Complete	17.922237	3.034713 $\times \;10^{-7}$
Trustworthy	20.460882	5.212000 $\times \;10^{-8}$
Predictable	23.807062	5.670811 $\times \;10^{-9}$
Safe-Reliable	15.016320	2.498807 $\times \;10^{-6}$
